# Transcutaneous Auricular Vagus Nerve Stimulation Enhances Emotional Processing and Long‐Term Recognition Memory: Electrophysiological Evidence Across Two Studies

**DOI:** 10.1111/psyp.70034

**Published:** 2025-03-11

**Authors:** Carlos Ventura‐Bort, Manon Giraudier, Mathias Weymar

**Affiliations:** ^1^ Department of Biological Psychology and Affective Science, Faculty of Human Sciences University of Potsdam Potsdam Germany; ^2^ Faculty of Health Sciences Brandenburg University of Potsdam Potsdam Germany

## Abstract

Recently, we found that continuous transcutaneous auricular vagus nerve stimulation (taVNS) facilitates the encoding and later recollection of emotionally relevant information, as indicated by differences in the late positive potential (LPP), memory performance, and late ERP Old/New effect. Here, we aimed to conceptually replicate and extend these findings by investigating the effects of different time‐dependent taVNS stimulation protocols. In Study 1, an identical paradigm to our previous study was employed with interval stimulation (30‐s on/off). Participants viewed unpleasant and neutral scenes on two consecutive days while receiving taVNS or sham stimulation and completed a recognition test 1 week later. Replicating previous results, unpleasant images encoded under taVNS, compared to sham stimulation, elicited larger amplitudes in an earlier window of the LPP during encoding, as well as more pronounced late Old/New differences. However, no effects of taVNS on memory performance were found. In Study 2, we followed up on these findings by synchronizing the stimulation cycle with image presentation to determine the taVNS effects for images encoded during the on and off cycles. We could replicate the enhancing effects of taVNS on brain potentials (early LPP and late Old/New differences) and found that taVNS improved recollection‐based memory performance for both unpleasant and neutral images, independently of the stimulation cycle. Overall, our results suggest that taVNS increases electrophysiological correlates of emotional encoding and retrieval in a time‐independent manner, substantiating the vagus nerve's role in emotional processing and memory formation, opening new venues for improving mnemonic processes in both clinical and non‐clinical populations.

## Impact statement

In two ERP studies we aimed to replicate and extend our previous findings on the enhancing effects of continuous taVNS on emotional processing and episodic memory retrieval by testing different stimulation protocols. Across studies we found that taVNS facilitated the initial processing (increased LPP amplitudes) and subsequent retrieval (larger Old/New effects) of unpleasant information. These results substantiate the vagus nerve's role in emotional processing and memory formation, which could also be relevant for clinical application.

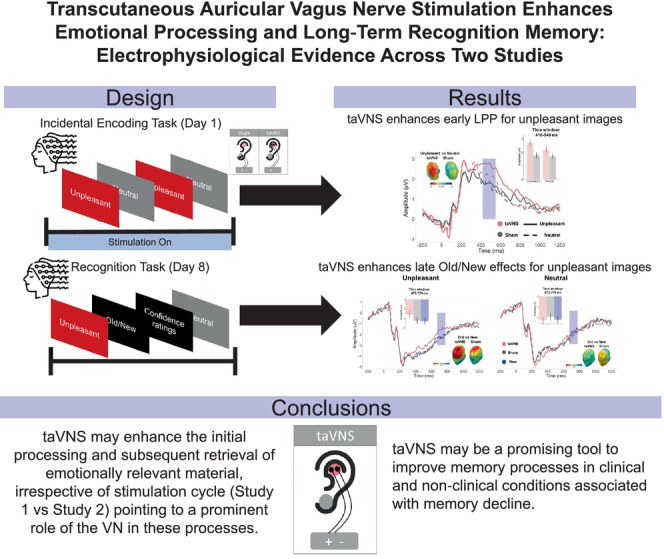

## 1 Introduction

There is ample evidence that emotionally salient information modulates different stages of processing, from earlier perceptual and attentional (e.g., Carretié 2014; Carretié et al. 2001, 2022) to later, more controlled processes (Bradley 2009; Hajcak and Foti 2020; Ribes‐Guardiola et al. 2023). The modulatory effects of emotion extend beyond the encoding episode, leading to more accurate long‐term episodic memories for emotional compared to neutral events (Bradley et al. 1992; Dolcos et al. 2017, 2020; Weymar and Hamm 2013). These emotion‐enhancing effects have been associated with the interaction between the release of peripheral (i.e., stress‐related) hormones –catecholamines and glucocorticoids– with central noradrenergic and glucocorticoid levels (McGaugh 2000; McIntyre et al. 2012; Schwabe 2017; Schwabe et al. 2022). Importantly, the vagus nerve (VN) has been identified as a key pathway through which peripheral catecholamine release can exert its effects (McIntyre et al. 2012). In a recent study, we could demonstrate for the first time in humans the involvement of the VN in the modulatory effects of emotion on memory (Ventura‐Bort et al. 2021). We observed that stimulating the VN using non‐invasive transcutaneous auricular VN stimulation (taVNS) favored the initial processing and subsequent retrieval of emotional information. In the current set of studies, we aimed to replicate and extend our previous findings by investigating the influence of time‐related parametrical variations of taVNS on initial processing and subsequent retrieval of emotional and neutral events.

Previous animal and human studies have reported that the amygdala (AMY), in interaction with other medial temporal lobe regions such as the hippocampus (HC), participates in the advantageous encoding of emotionally salient information (Dolcos et al. 2004, 2005; Kensinger and Corkin 2004; Sabatinelli et al. 2007). For instance, studies on the effects of emotion during encoding have reliably shown that viewing unpleasant and pleasant, compared to neutral images, produces larger AMY activation (Sabatinelli et al. 2005, 2013). Together with the AMY, regions of the inferotemporal, parietal, and occipital cortex also become more active when watching emotionally laden compared to neutral images (Sabatinelli et al. 2009, 2013). At an electrophysiological level, the enhanced activation of these regions during emotional processing has been related to the late positive potential (LPP; Liu et al. 2012; Sabatinelli et al. 2005, 2013). The LPP is a positive slow wave, maximal at central‐parietal electrode sites, that is reliably enhanced during the processing of both negative and positive stimuli compared to neutral ones (Schupp et al. 2000). This enhancement has been suggested to reflect the sustained engagement of attention to salient cues (Bradley 2009; Hajcak and Foti 2020, for a review see Lang and Bradley 2010).

It has been proposed that stress hormones secreted due to the encounter with an arousing event may facilitate the noradrenergic release in the AMY (McGaugh 2000, 2004; Roozendaal et al. 2009; Schwabe 2017; Schwabe et al. 2012), potentiating the enhancing effects of emotion. Supporting this view, it has been shown that greater cortisol levels are associated with larger AMY activation while viewing emotional vs. neutral scenes (Stegeren 2008). On the other hand, the intake of noradrenaline antagonists disrupts the increased AMY activity when processing emotionally relevant information both in humans (Schwabe et al. 2013; Strange and Dolan 2004; van Stegeren et al. 2008) and animals (Williams et al. 1998). Similar modulatory effects have been observed in LPP amplitudes. For instance, undergoing a stress induction protocol that promotes the release of adrenaline and cortisol potentiates the LPP increase for unpleasant compared to neutral images (Alomari et al. 2015; Weymar et al. 2012; but see Quaedflieg et al. 2013). Furthermore, LPP amplitudes may also be modulated by the intake of noradrenergic antagonists (de Rover et al. 2012; but see Weymar et al. 2010).

Extending these findings, it has reliably been found that the mnemonic advantage for emotionally relevant, relative to neutral, information is mediated by AMY activation in interaction with the HC, pinpointing their relevant role in the retrieval of emotional episodic memories (Dolcos et al. 2005; Ritchey et al. 2008). This emotion‐driven mnemonic advantage is mainly mediated by recollection, which, compared to familiarity, reflects an elaborate memory process that includes the storage and retrieval of additional spatial, temporal, or other contextual information (Dolcos et al. 2005; Ochsner 2000; Sharot et al. 2004). In line, event‐related potential (ERP) studies on memory retrieval also found that the recollection‐based memory advantage for emotional material is reflected in an enhanced late (> 400 ms) ERP Old/New effect (i.e., more positive/less negative waveforms for correctly identified old items compared to correctly detected new ones), which has been related to recollection‐based retrieval (Rugg and Curran 2007; Weymar and Hamm 2013). Notably, the long‐lasting memory‐enhancing effects of emotion have specially been found for longer retention intervals (Jaworek et al. 2014; Schaefer et al. 2011; Weymar et al. 2009; Wirkner et al. 2013, 2015), indicating that the recollection memory advantage for emotional events is facilitated by consolidation processes. As for the effects of emotion during the initial processing, the release of stress hormones during the encoding episode also modulates the behavioral and neural signature of emotional memories. For instance, administering noradrenergic antagonists during encoding diminished the subsequent recollection‐based memory advantage for emotional information (Cahill et al. 1994; Strange and Dolan 2004) and the associated hippocampal activation (Strange and Dolan 2004) and late ERP Old/New effects (Weymar et al. 2010). Similarly, acute stress protocols prior to the encoding phase have been shown to increase emotional memory performance (Cahill et al. 2003) and enhance the late ERP Old/New differences for emotional but not for neutral information (Wirkner et al. 2013).

It is important to note that the VN has been considered a critical path through which information about stress hormone release feeds back to the brain, influencing the activity in emotion‐ and memory‐sensitive brain regions via the nucleus of the solitary tract (NST) and locus coeruleus (LC) (Dorr and Debonnel 2006; Groves et al. 2005; Williams et al. 1998). The LC is considered a key node that participates in the neural amplification of relevant information during attentional selection and memory formation (Mather et al. 2016). Earlier animal studies established the causal role of ascending vagal fibers for emotional memory (Clark et al. 1995; Hulsey et al. 2017; McGaugh et al. 1993; McIntyre et al. 2012), but evidence in humans is rather scarce. In a first study, using invasive stimulation of the VN during the encoding of words, Clark and colleagues observed enhanced immediate memory performance for words (Clark et al. 1999; but see Mertens et al. 2022). Although these results may provide initial support for a role of the VN in memory in humans, the fact that emotional episodic memory was not tested constrains the generalization of the same neural path from animals to humans. Also, Clark et al. focused on immediate memory, but no evidence was provided as to whether the VN activation favors long‐term retention. In addition, Clark and colleagues used an invasive technique typically employed in patients suffering from specific disorders (e.g., epilepsy), limiting the possibilities to replicate the findings in healthy participants.

In order to address these voids and bring more insights into the role of the VN in emotional processing and episodic memory in healthy humans, in a recent study we investigated the influence of the VN activity—induced by non‐invasive taVNS—during encoding on long‐term emotional episodic memory (1‐week delay) (Ventura‐Bort et al. 2021). Previous findings suggest that taVNS may be a suitable tool for the current purpose, as it has been shown to modulate processes associated with the LC‐noradrenergic release (Burger et al. 2016, 2017; Szeska et al. 2020; Ventura‐Bort et al. 2018; but see Burger et al. 2018) and correlates of noradrenergic activation such as salivary alpha amylase (Giraudier et al. 2022). In our previous study, participants received continuous taVNS and sham stimulation on two consecutive days (in a counterbalanced fashion) while viewing a series of unpleasant and neutral pictures. One week after the first session, a recognition memory task took place in which the previously encoded images were intermixed with new ones, and participants were instructed to indicate whether the images were previously seen (i.e., old) or not (i.e., new) and how confident they were on their responses (to assess recollection and familiarity‐based recognition). During encoding, we found that taVNS, compared to sham, facilitated the discrimination between emotional and neutral scenes, as indicated by larger amplitude differences in an earlier time window of the LPP. During retrieval, we observed a recollection‐driven (trials with highest confidence) memory increase for emotional, but not neutral, images encoded under taVNS compared to sham. These findings were accompanied by larger recollection‐related brain potentials (late ERP Old/New differences) during retrieval of emotional scenes encoded under taVNS, compared to sham stimulation.

These results point to a role of the VN in the modulation of initial processing and subsequent retrieval of emotional information in humans and highlight the potential of taVNS as a neuromodulation technique to influence memory processes. Although these findings align with studies showing positive effects of taVNS on a variety of cognitive and affective processes (Burger et al. 2016; Fischer et al. 2018; Giraudier et al. 2020; Neuser et al. 2020; Poppa et al. 2022; Szeska et al. 2020; Ventura‐Bort et al. 2018; Ventura‐Bort and Weymar 2024; Villani et al. 2019), it should be noted that the beneficial effects of taVNS on these processes have not always been found (Burger et al. 2017; D'Agostini et al. 2022, 2025; Lucchi et al. 2024; Ludwig et al. 2025; Mertens et al. 2022; Warren et al. 2019). Therefore, to evaluate the consistency of these findings, direct or conceptual replication studies (Burger et al. 2017; Lloyd et al. 2023; Lucchi et al. 2024), meta‐ and mega‐analysis (Giraudier et al. 2022, 2024; Wolf et al. 2021), or studies empirically testing how stimulation‐related parametrical changes may affect the modulatory effects of taVNS are required (D'Agostini et al. 2023; Skora et al. 2024). For instance, research on the effects of taVNS on pupil dilation, as a potential physiological correlate of the LC‐NE system (Joshi et al. 2016; Murphy et al. 2011), has shown that taVNS compared to sham increases pupil dilation exclusively in response to the stimulation onset (D'Agostini et al. 2023; Lloyd et al. 2023; Sharon et al. 2021; Skora et al. 2024; Urbin et al. 2021; Wienke et al. 2023), but not during the whole stimulation period, suggesting that, at least for certain variables, the effects of taVNS may be time‐sensitive (e.g., phasic).

Considering the importance of taVNS replication studies (Lloyd et al. 2023) and building up on the potential effects of time‐dependent parameters, we therefore aimed at replicating our previous results by also varying time‐related taVNS conditions. In contrast to our previous study (Ventura‐Bort et al. 2021) in which active and sham stimulation were continuously applied throughout the encoding phase (i.e., all scenes were processed while delivering stimulation), in the first study we intended to replicate our previous findings; however, by using another stimulation duty cycle that alternates between on and off periods every 30 s (a default stimulation procedure most commonly employed in taVNS research; Farmer et al. 2021; Lucchi et al. 2024). Thus, we used the exact same design as in our previous study (Ventura‐Bort et al. 2021) except for changing the stimulation procedure from continuous to interval stimulation (30s on‐30s off cycle). If the effects of taVNS on emotional processing and retrieval are not dependent on the stimulation cycle, we expected to replicate our previous findings, showing enhanced initial processing (i.e., larger amplitudes in an early time window of the LPP) and subsequent recollection‐based retrieval (i.e., recollection‐driven memory performance and larger late ERP Old/New differences) for emotional scenes encoded under taVNS. However, if the effects of taVNS are particularly pronounced only for images encoded under actual concurrent stimulation (as in Ventura‐Bort et al. (2021), for all images under continuous stimulation), the effects might be less discernable during the current interval stimulation protocol (during which only about half of the images receive concurrent stimulation). In addition to the taVNS‐related effects, we expected to replicate previous basic emotion effects during passive viewing, as indicated by larger amplitudes for unpleasant compared to neutral images during an early and late time window of the LPP (Foti et al. 2009). Furthermore, during retrieval we also expected to replicate previous findings (Wilding and Ranganath 2012) showing overall ERP Old/New effects as indicated by significant differences between correctly identify old images compared to new ones, particularly for unpleasant information (e.g., Weymar et al. 2009, 2010).

## 2 Study 1

### 2.1 Methods

#### 2.1.1 Participants

Thirty‐one healthy students (27 women, 4 men; *M*_age_ = 21.32; SD_age_ = 4.05) from the University of Potsdam took part in the study in exchange for course credits or financial compensation. All participants had normal or corrected‐to‐normal vision and were native German speakers. Each individual provided written informed consent for a protocol approved by the Review Board of the German Psychological Society. Prior to the first session, participants were screened and invited to participate if they did not meet any of the following exclusion criteria: neurological or mental disorders, brain surgery, chronic or acute medication use, history of migraine and/or epilepsy: Data from one participant could not be analyzed due to technical problems related to the EEG recording, leaving a total of 30 participants (26 women; *M*_age_ = 21.26; SD_age_ = 4.01).

#### 2.1.2 Procedure and Tasks

We used the same procedure and design as in our previous publication (Ventura‐Bort et al. 2021), which consisted of a randomized, single‐blinded, within‐subject, cross‐over design (taVNS‐sham; sham‐taVNS). The experiment took place on three different days: two encoding sessions on two consecutive days and a retrieval session 1 week after the first encoding session.

We used a total of 240 images chosen from the *International Affective System* (IAPS; Lang and Bradley 2010) and from the *Nencki Affective Picture System* (NAPS; Marchewka et al. 2014). Images were preselected based on the standardized valence and arousal ratings and were grouped in a neutral (*N* = 120; e.g., buildings, neutral views, neutral human faces; *M*_valence_ = 5.12; SD_valence_; 0.35; *M*_arousal_ = 3.27; SD_arousal_ = 0.48) and an unpleasant category (*N* = 120, e.g., depicting mutilations, attacks, disgusting content, accidents; *M*_valence_ = 2.86; SD_valence_; 0.57; M_arousal_ = 5.5; SD_arousal_ = 0.86). Images were split into four different sets. Each set contained 30 neutral and 30 unpleasant images. Picture sets were assigned to the different conditions (old images encoded under taVNS, encoded under sham or used as new images) in a counterbalanced manner.

In each of the encoding sessions, 60 scenes (30 neutral, 30 unpleasant) were presented for 3000 ms with a varying inter‐trial interval (ITI) of 4000, 4500, or 5000 ms. Picture presentation was pseudorandomized with no more than two scenes of the same category presented in a row. Participants were instructed to attentively watch the pictures presented on the screen, and no mention of a later memory test was made (i.e., incidental encoding).

**FIGURE 1:**
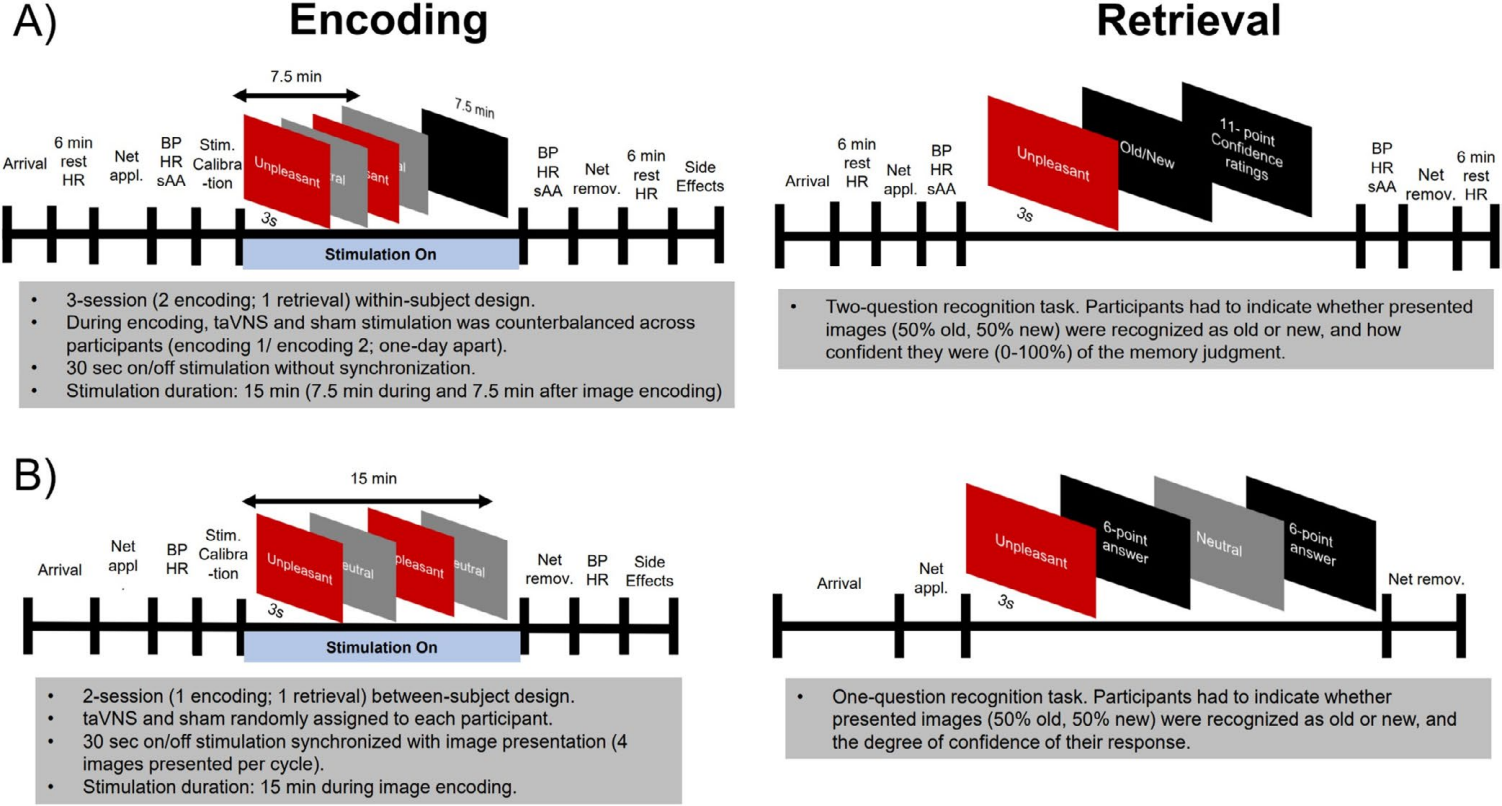
Schematic representation of the task design for Study 1 (A) and Study 2 (B).

Both incidental encoding sessions followed the same protocol (Figure 1). After arriving at the lab, participants sat on a comfortable chair in a dimly lit room. During the initial 6 min, heart rate activity was recorded at rest (data are reported elsewhere; Wolf et al. 2021). Following the resting period, the electroencephalography (EEG) net was applied, and different autonomic baseline measures were recorded and collected, including heart rate, blood pressure, and salivary samples to extract salivary‐alpha amylase (sAA) levels (i.e., putative marker of central noradrenergic activity). Thereafter, the stimulation electrodes were applied to the left ear and the intensity was adjusted (see procedure below). Following the stimulation calibration, participants performed the picture viewing task, which lasted approximately 7.5 min. To align the duration of stimulation with our previous study (Ventura‐Bort et al. 2021), participants were stimulated for an additional 7.5 min after picture encoding. Autonomic measures (heart rate, blood pressure, and sAA levels) were then recorded, the EEG net and the stimulation electrodes removed, and a second resting period of 6 min (including continuous heart rate measure) was performed without stimulation. Finally, subjective ratings on potential side effects were collected by asking participants, on a seven‐point scale (ranging from 1, *not at all*, to 7, *very much*), how strongly they experienced the following symptoms during the stimulation: headache, nausea, dizziness, neck pain, muscle contractions in the neck, stinging sensations under the electrodes, skin irritation in the ear, fluctuation in concentration or feelings, and unpleasant feelings.

Eight days after the first session, participants came back to the lab to perform a recognition memory task. After arrival, participants rested during a 6‐min interval while their heart rate was continuously recorded. Then the EEG electrodes were attached, a saliva sample was collected, and the recognition memory task was performed, which lasted approximately 26 min. During the recognition task, the 120 pictures encoded in the previous sessions (under taVNS and sham stimulation) were presented randomly intermixed with 120 new pictures. Each image was shown on the screen for 3000 ms, preceded by a 2000 ms fixation cross. Following picture offset, the question “Old/New?” was presented, and participants were instructed to make an old or new judgment. If the image was recognized as previously seen during the encoding sessions, they were instructed to press the ‘Old’ button on the keyboard, and if the image was identified as novel, they were instructed to press the “New” button. After button press, participants were instructed to rate the confidence of their recognition judgment on an 11‐point scale, ranging from 0 (not confident) to 100 (absolutely confident). Finally, autonomic measures and saliva samples were measured, the net was removed, and heart rate was continuously recorded again during a 6‐min resting period.

#### 2.1.3 Transcutaneous Auricular Vagus Nerve Stimulation (taVNS)

The taVNS stimulator, which consisted of two titan electrodes attached to a mount, was positioned in the left ear and wired to a stimulation unit (CMO2, Cerbomed, Erlangen, Germany). In the taVNS condition, the stimulator was placed in the left cymba conchae, an area innervated exclusively by the auricular branch of the vagus nerve (Ellrich 2019; Peuker and Filler 2002). As in previous studies using taVNS (D'Agostini et al. 2022; Giraudier et al. 2024; Ventura‐Bort and Weymar 2024), for the sham condition, the electrodes were positioned in the center of the left ear lobe, an area known to be free of vagal innervation (Ellrich 2019; Peuker and Filler 2002). The stimulation alternated between on and off phases every 30 s and was delivered with a pulse width of 250 μs at 25 Hz. The stimulation intensity was adjusted above the detection threshold and below the pain threshold. To individually set the stimulation intensity, participants received increasing and decreasing series of stimulation and rated the subjective sensation of the stimulation on a 10‐point scale, ranging from nothing (1), light tingling (3), strong tingling (6), to painful (10). The increasing series of trials started from an intensity of 0.1 mA and increased in 0.1 mA steps on a trial‐by‐trial basis until participants reported a sensation of 9. Before starting the decreasing series, the same intensity was repeated and then reduced trial by trial in 0.1 mA steps until a subjective sensation of 6 or below was experienced. This procedure was repeated a second time. The final stimulation intensity used for the experimental procedure was calculated based on the average of the 4 intensities rated as 8 (i.e., 2 from increasing and 2 from decreasing series). No differences were observed in the averaged intensity applied in the taVNS (*M*_intensity_ = 1.56, SD_intensity_ = 0.65) and in the sham conditions (*M*_intensity_ = 1.55, SD_intensity_ = 0.9); (*t* [29] = 0.029, *p* = 0.98). The interval stimulation was administered for approximately 15 min (7.5 min during encoding and 7.5 min after encoding).

#### 2.1.4 Autonomic Measures

To test the effects of stimulation on autonomic measures, including blood pressure (systolic and diastolic) and heart rate were measured before (baseline) and after stimulation with an upper arm cuff placed on the left arm using the Intelli Wrap Manschette M500 device (Omron Healthcare, Medizintechnik Handelsgesellschaft GmbH, Mannheim, Germany). In addition, salivary alpha‐amylase was also measured as a marker of endogenous noradrenergic activation. Saliva was collected by instructing participants to drool the saliva into a polypropylene tube. Saliva samples were stored at −20°C. Analyses were performed by the Dresden LabService GmbH (http://www.labservice‐dresden.de), using an enzyme kinetic method. Analysis of autonomic measures only comprised data collected during encoding, as we were exclusively interested in the modulatory effects of stimulation (taVNS vs. sham). Autonomic measures during retrieval were not analyzed.

#### 2.1.5 Electrophysiological Recording

EEG signals were recorded continuously from 129 electrodes using an Electrical Geodesics (EGI) HydroCel high‐density EEG system with NetStation software on a Macintosh computer. The EEG recording was digitized at a rate of 250 Hz, using the vertex sensor (Cz) as the recording reference. Scalp impedance for each sensor was kept below 50 kΩ, as recommended by the manufacturer guidelines. All channels were band‐pass filtered online from 0.1 to 250 Hz. Offline reduction was performed using *EEG Processing Open‐Source Scripts* (*EPOS*; Rodrigues et al. 2021) on *eeglab* (Delorme and Makeig 2004). In the first step, the EEG signal was re‐referenced to the average reference, bad electrodes were interpolated, and the data was filtered (lowpass filter of 20 Hz, highpass filter of 0.01 Hz), segmented (from 1 to 3 s after stimulus onset), and independent component analysis (ICA) performed. The *ICLabel* plug‐in was employed to flag potential artifactual components. In addition, we employed rigorous quality control procedures, including visual inspection of all components and verification of the flagged components. Only components clearly associated with artifacts were removed, minimizing the possibility of discarding genuine neural signals. The excluded components were saved. In the second step, the raw data were again filtered and segmented (from −200 to 1200 ms after stimulus onset) and the previously identified artifactual ICA components removed from the data. Subsequently, trials were baseline corrected (−200 to 0 ms before stimulus onset) and averaged based on the conditions of interest. For the encoding sessions, trials were grouped based on the *Affective Category* (unpleasant and neutral) for both the taVNS and sham conditions, separately. During the retrieval task, trials were grouped based on *Memory* (Old and New) as well as *Affective Category* and *Stimulation* (taVNS and sham).

### 2.2 Analyses

Analyses were performed in the *R* and *R Studio* environments. EEG analyses were performed using *MATLAB‐*based toolboxes.

#### 2.2.1 Self‐Report and Autonomic Measures

*T*‐tests comparing taVNS and sham stimulation for each reported subjective symptom were performed, separately. The effects of stimulation on autonomic reactivity and salivary levels were tested using repeated‐measures ANOVA with the within‐subject factors *Time* (pre‐ vs. post‐stimulation) and *Stimulation* (taVNS vs. Sham).

#### 2.2.2 Behavioral Performance^1^

##### 2.2.2.1 Recognition Memory

To evaluate the effects of taVNS on recognition memory performance, the discrimination index Pr, *p*(hit)‐ *p*(false alarm), was calculated for each stimulation condition and emotional category, separately. The Pr is an index of memory discrimination, with higher values associated with better discrimination. Only participants who could discriminate old versus new items were included in the analysis (overall Pr index > 0). All participants showed a Pr index larger than 0 and thus no participants had to be excluded. In addition, d‐prime^2^ derived from signal detection theory was extracted, *z*(*p[*hit]) − *z*(*p*[false alarm]), in order to favor comparisons with other studies. Pr and d‐prime were analyzed using linear mixed models, with the factors *Affective Category* (Unpleasant vs. Neutral) and *Stimulation* (taVNS vs. Sham) and their interaction as fixed factors. Participants intercepts were modeled as random effects. Significance was estimated with the R package *lmerTest*, which estimates the degrees of freedom using Satterthwaite approximation.

##### 2.2.2.2 Recognition Memory Based on Confidence Ratings

As for our previous study, we split memory performance based on the confidence rating to differentiate between familiarity and recollection processes. Recollection‐based memory indexes (both for Pr and d‐prime) were calculated based on trials with a confidence rating of 10, that is, *absolutely confident*, whereas Familiarity‐based memory was calculated considering trials with a confidence rating below 10. We decided on such memory split based on previous studies suggesting that familiarity‐based responses increase gradually as a function of recognition confidence, whereas recollection‐based responses are primarily associated with the highest level of confidence (Perfect et al. 1996; Rimmele et al. 2011; Weymar et al. 2009; Wixted and Stretch 2004; Yonelinas 2002). The modulating effects of taVNS on memory recognition based on confidence ratings were tested using linear mixed models with the factors *Affective Category*, *Stimulation*, and *Memory* (recollection‐ vs. familiarity‐based) and their interactions. Participants intercepts were modeled as random effects. Significance was estimated with the R package *lmerTest*, which estimates the degrees of freedom using Satterthwaite approximation.

Additionally, we used Bayes hypothesis testing analysis (Wagenmakers et al. 2018) to evaluate our hypotheses. Using a Bayesian approach encompasses the calculation of the predictive adequacy of two competing models to quantify the evidence provided by the data for one model over the other (Wagenmakers et al. 2018). To test the evidence in favor of the alternative hypothesis, we calculated the Bayes factor (BF_10_) on the significant effects by comparing final models to null models (i.e., models without the significant effects of interest). To interpret the results of the Bayes factors, the following classification was used (Lee and Wagenmakers 2013): a BF_10_ larger than 100 provides decisive evidence in favor of *H*_1_, a value between 30 and 100 indicates very strong evidence for *H*_1_, a score between 10 and 30 provides strong evidence for *H*_1_, a value between 3 and 10 indicates moderate evidence for *H*_1_, a value between 1 and 3 indicates anecdotal evidence for *H*_1_, and a BF_10_ of 1 provides no evidence for either *H*_1_ or *H*_0_. On the other hand, values between 0.3 and 1 provide anecdotal evidence for *H*_0_, values between 0.3 and 0.1 indicate moderate evidence for H_0_, scores between 0.1 and 0.03 show strong evidence for *H*_0_, values between 0.03 and 0.01 indicate very strong evidence for *H*_0_, and values lower than 0.01 provide decisive evidence for *H*_0_.

#### 2.2.3 Electrophysiology: Cluster‐Based Permutation Test

To test for stimulation effects on encoding and recognition‐related ERPs, data were submitted to a non‐parametric statistical testing procedure that includes correction for multiple comparisons (Maris and Oostenveld 2007), the so‐called cluster‐based permutation test. This test uses a two‐step procedure to identify significant effects between conditions. In a first step (i.e., sensor‐level criterion), *F*‐tests are performed for each time point and sensor. If significant effects (*p* < 0.05) that last for at least five consecutive time points (i.e., 20 ms) are detected, their *F*‐values are summed into the so‐called empirical “cluster masses.” In a second step (cluster‐level criterion), Monte Carlo simulations are used to draw 1000 permutations across experimental conditions and participants. Similar to with the empirical data, cluster masses are extracted from the permuted data, following the abovementioned criteria (i.e., *p* < 0.05 for at least 20 ms). To determine whether the empirical cluster masses reach significance, these are compared to the “critical cluster mass” defined as the 95th percentile of the permuted cluster masses. If the empirical cluster masses are bigger than the critical cluster mass, they are considered significant cluster masses.

##### 2.2.3.1 Encoding

Individual ERP averages were computed for each sensor, *Affective Category* (neutral and unpleasant), and *Stimulation* (taVNS and sham). Four participants were excluded due to bad quality data, leaving a total of 26 participants for the analysis. In line with our previous study (Ventura‐Bort et al. 2021; see also Bublatzky et al. 2020; Pastor et al. 2015; Rehbein et al. 2015), to test the interaction effects of stimulation on the processing of negative and neutral images, the *Affective Category* × *Stimulation* interaction was submitted to the cluster‐based permutation analysis that included posterior electrode sites within an earlier (200–600 ms) and later (600–1200 ms) time window. In addition to the interaction effects, the main effects of *Affective Category* and *Stimulation* were further tested (Supporting Information S1 and S2). We decided to split the time window of the LPP into an earlier and a later time window based on previous findings on emotional processing, demonstrating that the sustained positivity starting around 300 ms after stimulus onset may reflect different cognitive processes (Foti et al. 2009). For instance, Foti and colleagues (2009) observed that the initial portion of the LPP (between 300 and 600 ms after stimulus onset) was more consistent with a P3‐like component that reflects attentional processes (which was particularly influenced by taVNS, for example, Ventura‐Bort et al. 2018, 2021), whereas a later portion of the LPP (> 600 ms) may be associated with additional processes relevant for emotional processing. Thus, we considered that splitting the LPP into an earlier and later time window would help disentangle which of the two LPP subcomponents is rather modulated by taVNS.

##### 2.2.3.2 Retrieval

Individual ERP averages were computed for each sensor, *Affective Category* (neutral and unpleasant), and *Memory* (correctly identified old items encoded under taVNS, encoded under sham and correctly identified new items). Three participants were excluded due to bad quality data, leaving a total of 27 participants for the analysis. Although three factors were investigated during retrieval, including *Stimulation* (taVNS vs. sham), *Affective Category* (unpleasant vs. neutral), and *Memory* (old vs. new), our design was uneven as not all factors could fill each cell of the repeated‐measures design. This was due to the fact that we could not split between new images for the taVNS and sham conditions, as new images were the same for both conditions. To overcome this issue, we decided to first assess the interaction effects between *Affective Category* and *Stimulation* and thereafter investigate whether the significant interaction effects also reflected Old/New differences when compared to the new conditions (see for similar approach in Ventura‐Bort et al. 2021). Thus, based on our previous study in which interaction effects were observed in the late ERP Old/New effect, the cluster‐based permutation test was performed over both anterior and posterior electrode sites in the 400–1000 ms time window (which reflects the time epoch during which the Old/New effects are most prominent; Rugg and Curran 2007; Wilding and Ranganath 2012), for the *Affective Category* (neutral and unpleasant) and *Stimulation* (correctly identified old images under taVNS and under sham stimulation) interaction. If interaction effects emerged, ERP amplitudes were further compared to correctly identified new images to ensure that they reflected Old/New differences.

For both encoding and retrieval, significant interaction effects were followed by pairwise comparisons to unravel the nature of the interaction. In addition, Bayesian hypothesis testing of the significant interaction effects was carried out, using Bayesian ANOVAs on *JASP*. The model with the significant interaction effect was compared to a null model without such interaction, using participants' intercepts as random factors.

### 2.3 Results

#### 2.3.1 Self‐Report and Autonomic Measures

Results from the self‐reported symptoms after stimulation are shown in Table 1. Overall, reported symptoms were low and did not differ between taVNS and sham conditions in any of the screened symptoms (ps > 0.06), except for unpleasant feelings (*p* = 0.035).

**TABLE 1:** Mean subjective ratings (standard deviation) for the stimulation side effects (rated from 1, *not at all*, to 7, *very much*) in the active and sham condition (including t‐test comparing both conditions) in Study 1.

	taVNS	sham	*t*‐test
Headache	1.2 (0.48)	1.2 (0.55)	*t*_29_ = 0, *p* = 1
Nausea	1.13 (0.43)	1.03 (0.18)	*t*_29_1.14, *p* = 0.26
Dizziness	1.23 (0.77)	1.23 (0.56)	*t*_29_ = 0, *p* = 1
Neck pain	1.53 (0.9)	1.93 (1.20)	*t*_29_ = −1.75, *p* = 0.09
Neck contraction	1.87 (1.14)	2.17 (1.12)	*t*_29_ = −1.73, *p* = 0.1
Stinging sensation	1.93 (1.41)	1.63 (1.19)	*t*_29_ = 1.96, *p* = 0.06
Ear irritation	1.63 (1.27)	1.43 (0.77)	*t*_29_ = 0.95, *p* = 0.35
Concentration	2.47 (1.36)	2.48 (1.33)	*t*_29_ = −0.07, *p* = 0.94
Fluctuation of feelings	2.13 (1.43)	2.33 (1.54)	*t*_29_ = −1.44, *p* = 0.16
Unpleasant feelings	2.5 (1.61)	2.07 (1.39)	*t*_29_ = 2.21, *p* = 0.035

##### 2.3.1.1 Autonomic Measures

Mean heart rate, blood pressure, and sAA levels are reported in Table 2.

**TABLE 2:** Mean (standard deviation) of the autonomic and salivary measures before and after the stimulation in Study 1.

	Time	Heart rate (bpm)	Systolic blood pressure (mmHg)	Diastolic blood pressure (mmHg)	Alpha‐amylase (μkatal/L)	Log (alpha‐amylase)
taVNS	Pre	68.76 (9.98)	115.16 (12.03)	79.86 (6.91)	224.80 (131.88)	5.24 (0.60)
	Post	67.6 (8.59)	111.53 (8.38)	79.16 (6.92)	244.13 (150.12)	5.32 (0.60)
Sham	Pre	69.66 (9.42)	112.36 (12.2)	77.63 (6.83)	219.81 (157.04)	5.17 (0.67)
	Post	66.13 (9.72)	111.8 (9.26)	77.1 (6.85)	205.12 (136.80)	5.11 (0.69)s

For heart rate, a main effect of *Time* was found, *F*(1, 29) = 8.46, *p* = 0.007, *η*_p_^2^ = 0.23, indicating a higher heart rate before compared to after encoding, but neither *Stimulation*, *F*(1, 29) = 0.04, *p* = 0.83, *η*_p_^2^ = 0.0, nor interaction effects reached significance, *F*(1, 29) = 2.25, *p* = 0.12, *η*_p_^2^ = 0.07.

For systolic blood pressure, a main effect of *Time* was observed, *F*(1, 29) = 4.19, *p* = 0.049, *η*_p_^2^ = 0.12, indicating higher systolic blood pressure before compared to after the encoding phase, but no *Stimulation*, *F*(1, 29) = 1.18, *p* = 0.28, *η*_p_^2^ = 0.04, or *Time* × *Stimulation* interaction effects were found, *F*(1, 29) = 3.08, *p* = 0.089, *η*_p_^2^ = 0.1.

For diastolic blood pressure, no main effect of *Time* was observed, *F*(1, 29) = 0.76, *p* = 0.39, *η*_p_^2^ = 0.03, but a significant effect of *Stimulation* was found, *F*(1, 29) = 4.64, *p* = 0.04, *η*_p_^2^ = 0.14, indicating overall higher diastolic blood pressure under taVNS than sham stimulation. Nevertheless, no significant *Time* × *Stimulation* interaction effects were observed, *F*(1, 29) = 0.01, *p* = 0.9, *η*_p_^2^ = 0.0.

For sAA levels, no main effects of *Time*, *F*(1, 27) = 0.10, *p* = 0.75, *η*_p_^2^ = 0.00, *Stimulation*, *F*(1, 27) = 0.98, *p* = 0.33, *η*_p_^2^= 0.03, or interaction were observed, *F*(1, 27) = 1.92, *p* = 0.17, *η*_p_^2^ = 0.06.

#### 2.3.2 Behavioral Performance

Table 3 summarizes the behavioral results (mean and standard deviation) for the recognition memory task as a function of *Affective Category* and *Stimulation*.

**TABLE 3:** Mean (standard deviation) of behavioral indices for unpleasant and neutral scenes encoded under sham and taVNS stimulation in Study 1.

	Sham		taVNS	
	Unpleasant	Neutral	Unpleasant	Neutral
Item recognition				
Pr	0.58 (0.15)	0.51 (0.16)	0.58 (0.12)	0.47 (0.15)
d’	1.82 (0.55)	1.59 (0.61)	1.82 (0.50)	1.48 (0.49)
Recognition memory based on confidence				
Familiarity‐based PR	0.16 (0.22)	0.17 (0.20)	0.13 (0.21)	0.17 (0.18)
Recollection‐based PR	0.38 (0.21)	0.30 (0.20)	0.40 (0.19)	0.27 (0.19)
Familiarity‐based d’	0.71 (0.61)	0.77 (0.64)	0.64 (0.61)	0.75 (0.51)
Recollection‐based d’	2.01 (0.83)	1.74 (0.98)	2.00 (0.97)	1.57 (0.108)

##### 2.3.2.1 Recognition Memory

For the Pr index, a significant effect of *Affective Category* was found, *t*(87) = 2.81, *p* = 0.006, indicating better memory for unpleasant compared to neutral scenes. However, no *Stimulation*, *t*(87) = −1.43, *p* = 0.15, or interaction effects were observed, *t*(87) = 0.98, *p* = 0.32. These results were confirmed by Bayes hypothesis testing analysis, as indicated by very strong evidence for an effect of *Affective Category*, BF_10_ = 38.45, and moderate evidence for a lack of a *Stimulation*, BF_10_ = 0.25, and *Affective Category* × *Stimulation* interaction effects, BF_10_ = 0.28 (see Section S9 of the Supporting Information).

Linear mixed models on d‐prime revealed a significant effect of *Affective Category*, *t*(87) = 2.55, *p* = 0.01, indicating better memory performance for unpleasant compared to neutral images, but no *Stimulation*, *t*(87) = −1.32, *p* = 0.19, or interaction effects, *t*(87) = 0.09, *p* = 0.36. Bayes hypothesis testing analysis revealed moderate evidence for an effect of *Affective Category*, BF_10_ = 8.22, and moderate evidence for a lack of *Stimulation*, BF_10_ = 0.23, or interaction effects, BF_10_ = 0.23.

##### 2.3.2.2 Recognition Memory Based on Confidence Ratings

**FIGURE 2:**
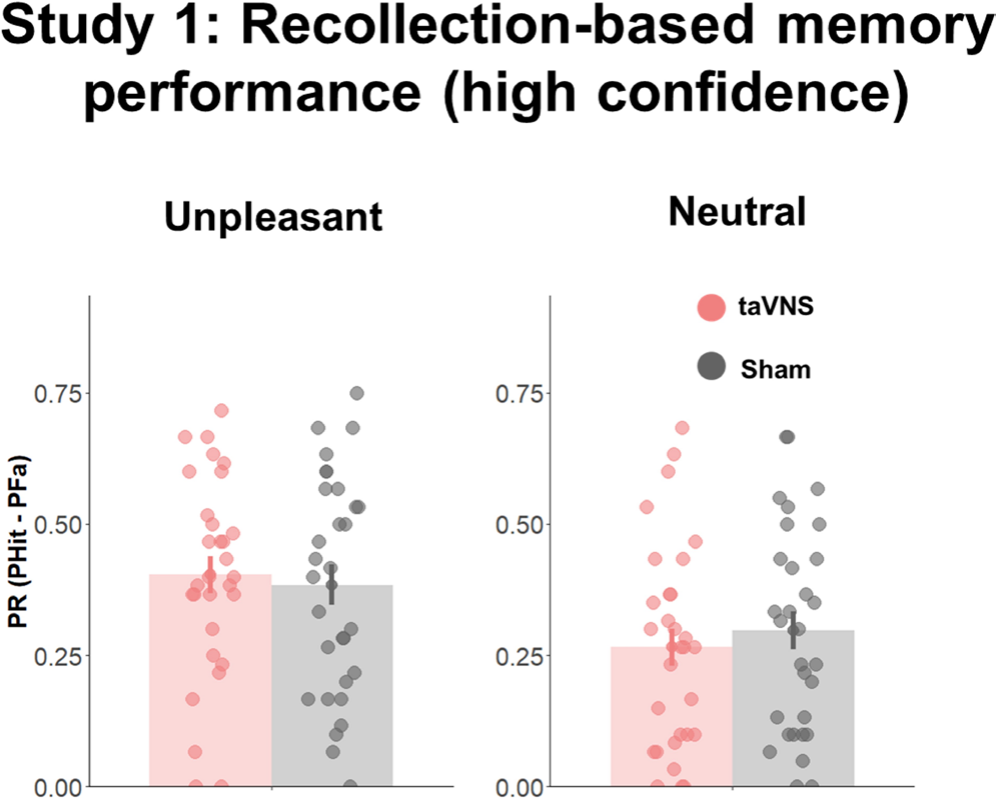
Memory performance for recollection‐related (highest confidence) judgments. Depiction of behavioral performance for unpleasant and neutral images in Study 1. Error bars represent standard error. Bar plots represent mean values. Dots indicate individual scores.

When memory performance was split based on subjective confidence ratings in recollection (rating = 10) and familiarity‐based (rating: 1–9), for the Pr index we observed no significant effects of *Memory*, *t*(232) = 1.76, *p* = 0.08, *Affective Category*, *t*(232) = −0.26, *p* = 0.79, or *Stimulation*, *t*(232) = −0.119, *p* = 0.91. Moreover, none of the interaction effects approached significance (*Affective Category* × *Memory*, *t*(232) = 1.50, *p* = 0.13; *Affective Category* × *Stimulation*, t(232) = −0.22, *p* = 0.82; *Memory* × *Stimulation*: *t*(232) = −0.41, *p* = 0.68; *Affective Category* × *Stimulation* × *Memory*: *t*(203) = 0.71, *p* = 0.48). Despite the lack of significant effects, Bayes hypothesis testing revealed decisive evidence for *Memory* effects, BF_10_ > 100, suggesting larger recollection than familiarity‐based memory performance, and moderate evidence for an *Affective Category* × *Memory* interaction, BF_10_ = 7.05. No evidence for the remaining tested effects was found (BF_10s_ < 0.87; Figure 2).

For the d‐prime index, we found an effect of *Memory*, *t*(203) = 4.80, *p* < 0.001, indicating that memory was more driven by recollection than familiarity, but no effect of *Affective Category*, *t*(203) = −0.31, *p* = 0.75, or *Stimulation* was obtained, *t*(203) = −0.10, *p* = 0.92. Moreover, none of the interactions reached significance (*Affective Category* × *Memory*, *t*(203) = 1.14, *p* = 0.25; *Affective Category* × *Stimulation*, *t*(203) = −0.15, *p* = 0.88; *Memory* × *Stimulation*: *t*(232) = −0.54, *p* = 0.58; *Affective Category* × *Stimulation* × *Memory*: *t*(203) = 0.51, *p* = 0.61). Bayes hypothesis testing confirmed these findings, as indicated by decisive evidence for *Memory* effects, BF_10_ > 100, but moderate to strong evidence against all other effects (BF_10s_ < 0.29), except for the *Affective Category* × *Memory* interaction, which showed anecdotal evidence (BF_10_ = 1.51).

#### 2.3.3 Electrophysiological Analyses: Permutation Test

**FIGURE 3:**
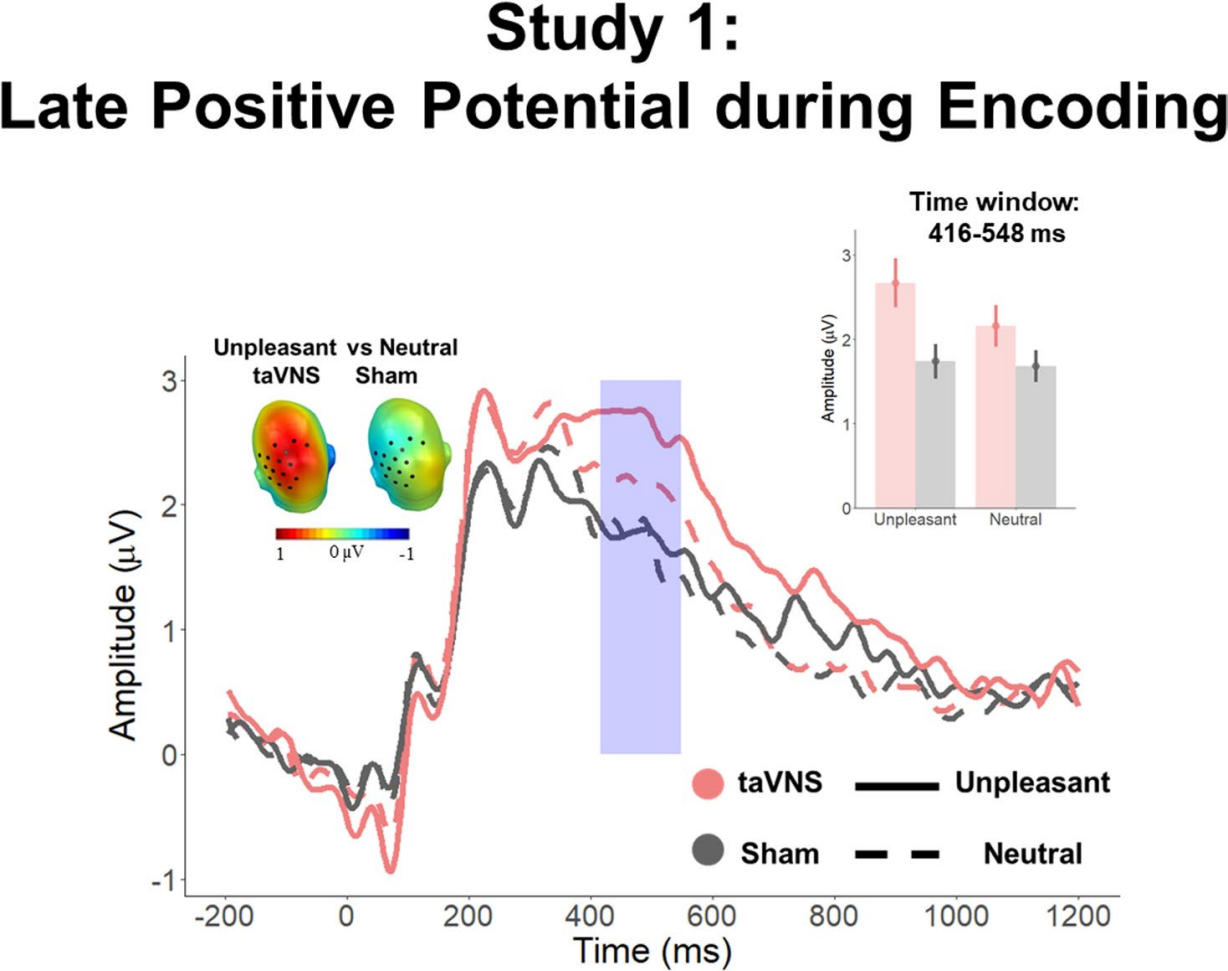
ERP results during encoding in Study 1. ERP‐averaged waveforms across the significant sensor clusters. The blue box indicates the significant time window. Upper right inset: Mean averaged ERPs during the significant time window and sensor clusters, showing a larger emotion discrimination during taVNS than sham condition. Upper left inset: Scalp topographies showing emotional differences during the taVNS and sham conditions, separately.

**FIGURE 4:**
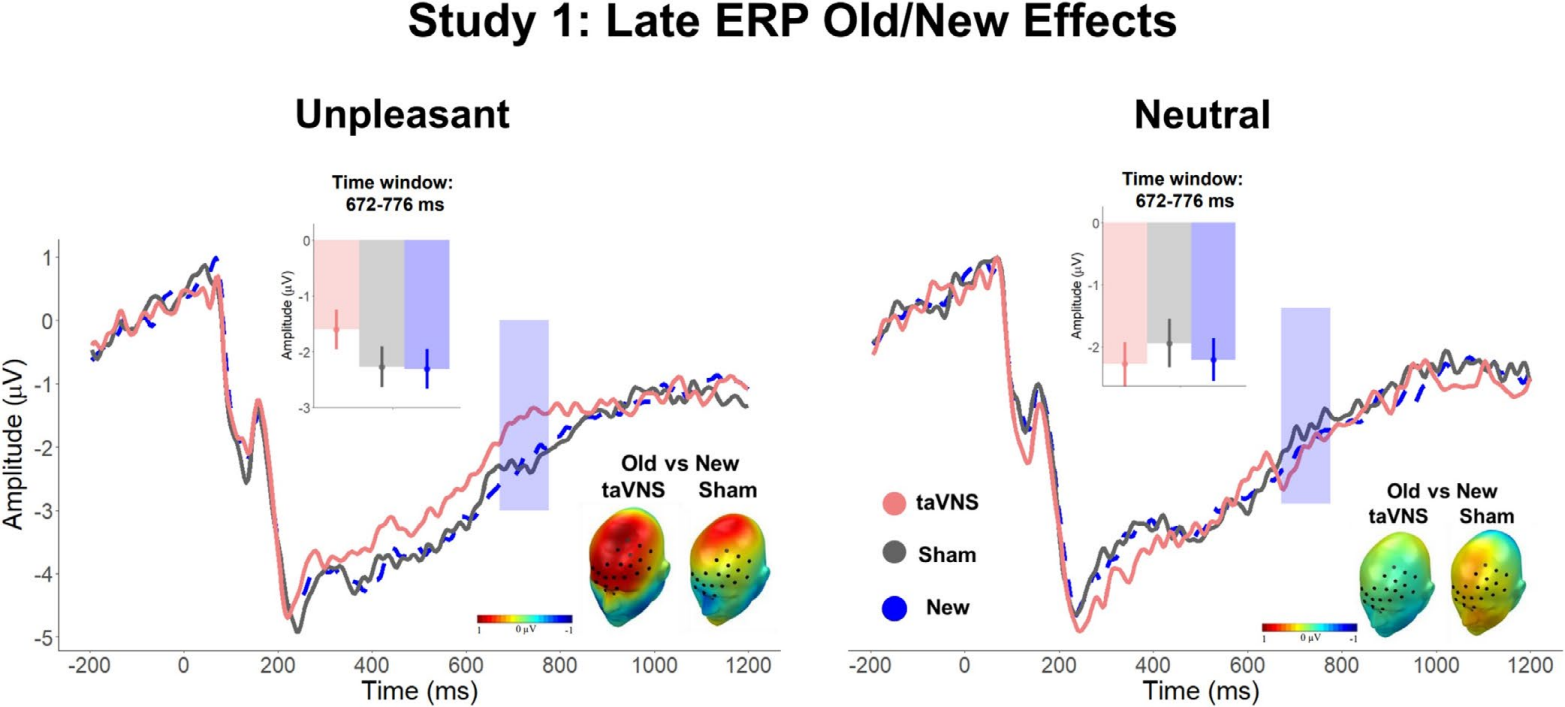
ERP results during retrieval in Study 1. ERP‐averaged waveforms across the significant sensor cluster (left for unpleasant images and right for neutral images). The blue box indicates the significant time window. Upper inset: Mean averaged ERPs during the significant time window (672–776 ms) and sensor cluster, showing a larger activity for correctly retrieved unpleasant images encoded during taVNS, compared to sham. Lower inset: Topographical Old/New differences for taVNS and sham conditions, separately.

Figures 3 and 4 depict the main *Affective Category* × *Stimulation* interaction effects found during encoding and recognition, respectively.

##### 2.3.3.1 Encoding

Cluster‐based permutation tests on the main effects of *Affective Category* and *Stimulation* are reported in Sections S1 and S2 of the Supporting Information.

Cluster‐based permutation tests on the interaction effects of *Affective Category and Stimulation* revealed that in the early time window (200–600 ms) 4 clusters in the posterior region surpassed the cluster mass of 756.5 (Cluster 1: mass = 2377.1, time window = 416–548 ms, sensors = 37, 41, 42, 47, 51, 52, 53, 54, 55, 59, 60, 61, 66, 67, and 79; Cluster 2: mass = 1366.1, time window = 404–544 ms, sensors = 100, 101, 102, 107, 108, and 113; Cluster 3: mass = 1137.5, time window = 208–248 ms, sensors = 45, 50, 56, 57, 58, 63, 64, 65, 68, 69, 73, 74, 87, 82, 88, 89, 94, 95, 99, and 107; Cluster 4: 1184.7, time window = 324–368 ms, sensors = 91, 92, 93, 95, 96, 97, 98, 99, 100,101, 102, 103, 107, and 108).

In line with our previous study, analysis on Cluster 1 revealed a main effect of *Affective Category*, *F*(1, 25) = 7.71, *p* = 0.01, *η*_p_^2^ = 0.23, a main effect of *Stimulation*, *F*(1, 25) = 6.31, *p* = 0.02, *η*_p_^2^ = 0.20, and an interaction *Affective Category* × *Stimulation*, *F*(1, 25) = 10.89, *p* = 0.003, *η*_p_^2^ = 0.30 (BF_10_ = 1.48). Subsequent *t*‐tests revealed significant differences between neutral and unpleasant scenes encoded under taVNS, *t*(26) = −4.10, *p* < 0.001, but not under sham, *t*(26) = 0.59, *p* = 0.61. Similarly, differences between taVNS and sham stimulation emerged for unpleasant images, *t*(26) = −3.19, *p* = 0.002, but not for neutral ones, *t*(26) = −1.13, *p* = 0.27 (Figure 3; Supporting Information S3 for results on the other clusters).

In the late time window (600–1200 ms), one posterior cluster surpassed the cluster mass of 568.5 (Cluster 1: mass = 642.1, time window = 672–724 ms, sensors = 37, 53, 54, 55, 61, 62, 78, 79, and 87).

Results revealed a main effect of *Affective Category*, *F*(1, 25) = 27.46, *p* < 0.001, *η*_p_^2^ = 0.52, a main effect of *Stimulation*, *F*(1, 25) = 4.43, *p* = 0.046, *η*_*p*_^*2*^ = 0.15, and an interaction *Affective Category* × *Stimulation*, *F*(1, 25) = 10.04, *p* = 0.004, *η*_p_^2^ = 0.29 (BF_10_ = 6.38). Subsequent *t*‐tests revealed significant differences between neutral and unpleasant images encoded under taVNS, *t*(26) = −5.91, *p* < 0.001, but not under sham, *t*(26) = −1.73, *p* = 0.09. Moreover, differences between taVNS and sham for unpleasant images were observed, *t*(26) = −3.78, *p* < 0.001, but not for neutral ones, *t*(26) = 0.25, *p* = 0.81.

##### 2.3.3.2 Retrieval

Four clusters surpassed the cluster mass of 995.5 (Cluster 1: mass = 4504.7, time window: 656–776 ms, sensors = 59, 60, 62, 65, 66, 67, 69, 70, 71, 72, 73, 74, 75, 76, 77, 78, 79, 81, 82, 83, 84, 85, 86, 88, 89, 90, 91, 92, 94, 95, 96, 97, 98, 99, 101, and 102; Cluster 2: mass = 2215.1, time window: 672–776 ms, sensors = 3, 9, 10, 11, 12, 13, 14, 15, 16, 16, 18, 19, 20, 21, 22, 23, 24, 27, 28, 29, 34, and 35; Cluster 3: mass: 2055.3, time window: 400–480 ms, sensors = 13, 20, 27, 28, 29, 30, 31, 33, 34, 35, 36, 37, 39, 40, 41, 42, 45, 46, and 47; Cluster 4: mass = 1171.9, time window = 408–476 ms, sensors = 94, 95, 96, 99, 100, 101, 102, 107, 108, 109, 113, 115, 121, 125, and 126).

Analysis on Cluster 1 (time window: 656–776 ms) revealed neither a main effect of *Affective Category*, *F*(1, 26) = 0.45, *p* = 0.83, *η*_p_^2^ = 0.0, nor a main effect of *Stimulation*, *F*(1, 25) = 0.15, *p* = 0.90, *η*_p_^2^ = 0.0, but an interaction *Affective Category* × *Stimulation* emerged, *F*(1, 25) = 10.0, *p* = 0.004, *η*_p_^2^ = 0.28 (BF_10_ = 11.69). To ensure that the interaction effects reflected memory‐related processes, the significant effects were further compared to the amplitudes evoked by the correctly identified new material. However, the expected Old/New differences were not observed: New Unpleasant versus Old Unpleasant taVNS: *t*(26) = 1.88, *p* = 0.21; New Unpleasant versus Old Unpleasant sham: t(26) = 0.42, *p* = 0.99; Old Unpleasant taVNS versus Old Unpleasant sham: *t*(26) = 1.87, *p* = 0.22; New Neutral versus Old Neutral taVNS: *t*(26) = −0.74, *p* = 1; New Neutral versus Old Neutral sham: *t*(26) = 2.91, *p* = 0.02; Old Neutral taVNS vs. Old Neutral sham: *t*(26) = 3.09, *p* = 0.014.

For Cluster 2 (time window: 672–776 ms), no main effect of *Affective Category*, *F*(1, 26) = 0.58, *p* = 0.45, *η*_p_^2^ = 0.02, or *Stimulation* were observed, *F*(1, 26) = 0.73, *p* = 0.40, *η*_p_^2^ = 0.03, but the *Affective Category* × *Stimulation* interaction reached significance, *F*(1, 26) = 9.92, *p* = 0.004, *η*_p_^2^ = 0.27 (BF_10_ = 6.77). Follow‐up analysis revealed that old unpleasant images encoded under taVNS produced a less negative deflection than correctly identified new unpleasant images, *t*(26) = 2.2, *p* = 0.037, and old unpleasant images encoded under sham, *t*(26) = 2.32, *p* = 0.028. No other comparison reached significance (New Unpleasant vs. Old Unpleasant sham: *t*(26) = 0.42, *p* = 0.67; New Neutral vs. Old Neutral taVNS: *t*(26) = 0.48, *p* = 0.63; New Neutral vs. Old Neutral sham: *t*(26) = −1.89, *p* = 0.07; Old Neutral taVNS vs. Old Neutral sham: *t*(26) = −1.66, *p* = 0.11; Figure 4).

For Cluster 3 (time window 400–480 ms), no main effect of *Affective Category* was observed, *F*(1, 26) = 2.11, *p* = 0.16, *η*_p_^2^ = 0.08, or *Stimulation F*(1, 25) = 0.059, *p* = 0.81, *η*_p_^2^ = 0.0, but the *Affective Category* × *Stimulation* interaction reached significance, *F*(1, 26) = 16.07, *p* < 0.001, *η*_p_^2^ = 0.38 (BF_10_ = 20.15). Subsequent testing showed that old unpleasant images encoded under taVNS produced a less negative deflection than correctly identified new unpleasant images, *t*(26) = 2.61, *p* = 0.015, and old unpleasant images encoded under sham, *t*(26) = 2.69, *p* = 0.012. Moreover, neutral images encoded under taVNS produced smaller amplitudes than neutral images encoded under sham: *t*(26) = −2.70, *p* = 0.012. No other comparison approached significance (New Unpleasant vs. Old Unpleasant sham: *t*(26) = 0.19, *p* = 0.85 New Neutral vs. Old Neutral taVNS: *t*(26) = 1.90, *p* = 0.07; New Neutral vs. Old Neutral sham: *t*(26) = −1.26, *p* = 0.22).

For Cluster 4 (time window: 408–476 ms), a main effect of *Affective Category* was observed, *F*(1, 26) = 5.10, *p* = 0.03, *η*_p_^2^ = 0.16. In the absence of a *Stimulation* effect, *F*(1, 26) = 1,74, *p* = 0.19, *η*_p_^2^ = 0.08, an interaction *Affective Category* × *Memory* was found, *F*(1, 26) = 12.27, *p* = 0.002, *η*_p_^2^ = 0.32 (BF_10_= 31.028). Post hoc analysis revealed that old unpleasant images encoded under sham produced larger activity than unpleasant images encoded under taVNS, *t*(26) = −3.23, *p* = 0.003. Additionally, new unpleasant images produced larger activity than old unpleasant images encoded under taVNS: *t*(26) = 2.19, *p* = 0.03; No other contrasts reached significance: New Unpleasant vs. Old Unpleasant sham: *t*(26) = −1.73, *p* = 0.09; New Neutral versus Old Neutral taVNS: *t*(26) = −1.71, *p* = 0.09; New Neutral vs. Old Neutral sham: *t*(26) = 0.61, *p* = 0.54; Old Neutral taVNS vs. Old Neutral sham: *t*(26) = 1.92, *p* = 0.06.

Altogether, cluster‐based permutation tests revealed two memory‐related significant interaction effects (Cluster 2 and Cluster 3) over frontal and central regions in the 672–776 ms and 400–480 ms time windows that were characterized by increased Old/New differences for unpleasant scenes encoded under taVNS compared to sham stimulation. However, no memory‐related interaction effects were observed for the neutral scenes.

### 2.4 Discussion

This study aimed to replicate and extend previous results showing improving effects of taVNS on encoding and retrieval of emotional and neutral material by also testing its impact when using a different stimulation cycle. Employing an identical design to our previous study, we applied a 30 s on/off interval stimulation protocol—instead of a continuous stimulation protocol (Ventura‐Bort et al. 2021). During encoding, the processing of emotional images evoked larger amplitudes than their neutral counterparts, particularly in the late time window of the LPP (see Supporting Information S1 and S8), replicating previous findings (e.g., Hajcak and Foti 2020; Schupp et al. 2000). Furthermore, we could find interaction effects of stimulation on the processing of emotional and neutral images. Specifically, in an earlier (416–548 ms) and later (672–724 ms) time window of the LPP, we did observe enhanced amplitudes while processing emotional vs. neutral information under taVNS compared to sham stimulation. Similar interaction effects were observed during memory retrieval, revealing that unpleasant images previously encoded under taVNS elicited larger ERP Old/New differences than unpleasant images encoded under sham (672–776 ms), in line with our previous findings (Ventura‐Bort et al. 2021). However, unlike our previous report, we were unable to observe the recollection‐driven advantage for unpleasant images encoded under taVNS on memory performance.

The fact that the behavioral results could not be replicated across studies suggests that the enhancing effect of taVNS on memory performance may be dependent on the stimulation protocol used. It could be that the usage of interval stimulation lowered the effects of taVNS on memory, impeding to observe enhancing effects at a behavioral level. This assumption is based on previous investigations into the effects of taVNS on pupil dilatation in humans, showing increased amplitudes exclusively linked to the stimulation onset (Capone et al. 2021; Lloyd et al. 2023; Sharon et al. 2021; Skora et al. 2024; Urbin et al. 2021; Wienke et al. 2023) but not when the overall task or stimulation period was considered (Burger et al. 2020; Keute et al. 2019; Warren et al. 2019). VNS time‐dependent effects have also been reported in animal studies in which VNS has been shown to be most efficient when synchronized with task‐relevant events (Dorr and Debonnel 2006; Ganzer et al. 2018; Groves et al. 2005; Mridha et al. 2021).

To systematically assess the differences between on and off stimulation intervals in our design, it would be necessary to distinguish between images encoded during each cycle. However, in the current study, we could not accurately identify which images were encoded during the on and off periods. This limitation was overcome in our second study, in which image presentation was synchronized with the stimulation cycles, allowing us to separate between images encoded during on and off stimulation phases.

## 3 Study 2

### 3.1 Introduction

In this second study, we followed up on the results of Study 1 by testing more specifically the effects of stimulation cycle on emotional processing and retrieval. To this end, we used a similar design to our prior study, in which participants encoded unpleasant and neutral images while receiving interval stimulation. However, this time, the image presentation was synchronized with the cycles of the stimulation, allowing us to identify which images were presented during the on and during the off cycles. This characteristic permits us to determine whether the absence of taVNS effects on memory performance in Study 1 was dependent on the stimulation cycle. The addition of a *Stimulation Phase* factor led to a two‐session mixed design in which participants were randomly assigned to either a taVNS or sham group^3^. In both groups, during the encoding session, images were alternately encoded during on and off cycles. As in Study 1, participants underwent a recognition test (1 week later), in which the encoded images were intermixed with new ones.

Given that in Study 1 we could replicate the effects of taVNS at an electrophysiological level, we hypothesized that the stimulus‐synchronized stimulation cycle would not alter the enhancing effects of taVNS on the ERPs. For memory performance, however, if the synchronization of the stimulation with task‐relevant events is critical for memory formation, we expected to observe improvements as a function of stimulation phase. More specifically, we hypothesized that taVNS may facilitate the memory retrieval of images encoded during the on cycle.

### 3.2 Methods

#### 3.2.1 Participants

A total of 65 healthy students (49 women, 13 men; 3 diverse; *M*_age_ = 24.24; SD_age_ = 5.25) from the University of Potsdam participated in the study in exchange for course credits or financial compensation. All participants had normal or corrected‐to‐normal vision and were native German speakers. Each individual provided written informed consent for a protocol approved by the Review Board of the German Psychological Society. Prior to the lab session, participants were screened online and invited to participate if they did not meet any of the exclusion criteria (see Study 1).

#### 3.2.2 Procedure and Tasks

In Study 2, a randomized, single‐blinded, mixed design was used. Participants were either assigned to the taVNS or sham group. The 2‐day experiment consisted of an encoding session and a retrieval session that took place 1 week later.

We used a total of 240 images chosen from the *International Affective System* (IAPS; Lang and Bradley 2010) from the *Nencki Affective Picture System* (NAPS; Marchewka et al. 2014) and from the *EmoMadrid Set* (Carretié et al. 2019). Images were preselected based on the standardized valence and arousal ratings and were grouped in a neutral (*N* = 120; e.g., buildings, neutral views, neutral human faces; *M*_valence_ = 5.23; SD_valence_; 0.35; *M*_arousal_ = 4.40; SD_arousal_ = 0.84) and an unpleasant category (*N* = 120, e.g., depicting mutilations, attacks, disgusting content, accidents; *M*_valence_ = 2.30; SD_valence_; 0.71; *M*_arousal_ = 7.29; SD_arousal_ = 0.98). Pictures were counterbalanced across participants by creating eight different lists (for similar procedure, see Ventura‐Bort, Dolcos et al. 2020; Ventura‐Bort et al. 2016, 2019, 2020; Ventura‐Bort, Wendt, et al. 2020). In each list, images were arranged in four sets (30 unpleasant and 30 neutral images). Sets were comparable in terms of valence, arousal, and content. Image sets were assigned to the ON condition (e.g., set 1), OFF condition (e.g., set 2), or were used as novel images for retrieval (e.g., sets 3 and 4). Each of the eight sets was equally assigned to each of the experimental conditions across lists. Each participant was randomly assigned to one of the eight lists.

During encoding (Figure 1), 120 images (60 neutral, 60 unpleasant) were presented for 3000 ms with a varying inter‐trial interval (ITI) between 2500 and 5000 ms. Picture presentation was pseudorandomized, with no more than two scenes of the same category presented consecutively. To ensure that the presentation of the images matched the 30‐s on and 30‐s off stimulation cycles, image presentation was manually synchronized with the stimulator, so that four images were presented alternately during the on cycle and four images during the off cycle. Specifically, the start of the task and the stimulator were manually synchronized. During the first minute, no image was presented while participants received a full cycle of stimulation (30 s on and 30 s off). Then, the image presentation started (synchronized with on‐stimulation cycle). It should be noted that the refreshing rate of the screen (60 Hz) causes slight delays in the presentation of images that accumulate throughout the task. We therefore continuously adjusted the ITIs between cycles to avoid asynchronicity between stimulation and image presentation across the task. During the task, participants were instructed to attentively look at the pictures presented on the screen. No mention of a later memory test was made (i.e., incidental encoding).

After arrival, participants sat on a comfortable chair in a dimly lit room. Before starting the task, various autonomic baseline measures were recorded, including heart rate, blood pressure, and salivary samples. The stimulation electrodes were then applied to the left ear, and the intensity was adjusted (see Transcutaneous auricular vagus nerve stimulation section of Study 1). No differences were observed between the averaged intensity that was applied in the taVNS (*M*_intensity_ = 1.33, SD_intensity_ = 0.74) and in the sham group (*M*_intensity_ = 1.32, SD_intensity_ = 0.79); (*t* [63] = −0.49, *p* = 0.61). Following the stimulation calibration, the electroencephalography (EEG) net was applied, and participants performed the encoding task, which lasted approximately 15 min. Thereafter, the EEG net and the stimulation electrodes were removed, and autonomic measures (heart rate, blood pressure) recorded. At the end of the session, participants completed a questionnaire on potential side effects (see Study 1).

One week after the encoding session, participants came back to the lab to perform a recognition memory task. After arrival, the EEG electrodes were attached, and the recognition memory task was conducted, which lasted approximately 26 min. During the recognition task, the 120 encoded pictures (encoded during on and off stimulation periods) were presented randomly intermixed with 120 new pictures. Each image was shown on the screen for 3000 ms, preceded by a 2000 ms fixation cross. Following picture offset, recognition memory was tested using a 6‐point scale to which participants were asked to indicate whether the image was old or new and how confident they were in their answers. The six alternatives were as follows: (1) the picture is definitely new, (2) it is probably new, (3) it is perhaps new, (4) it is perhaps old, (5) it is probably old, and (6) it is definitely old. It should be noted that the response alternatives in the recognition memory task changed in Study 2 compared to Study 1, in which we used a two‐question task (i.e., Old/New judgment, and Confidence rating judgment). For Study 2, we decided to combine both in one single question for two main reasons: (1) Simplifying the recognition task to make it more efficient and (2) facilitating the comparison with other studies by using response anchors that have been more commonly used in memory research (Yonelinas 2002). The taVNS device, autonomic measures, and electrophysiological recording were identical to Study 1.

### 3.3 Analyses

Analyses were performed in the *R* and *R Studio* environments. EEG analyses were performed using *MATLAB‐*based toolboxes.

#### 3.3.1 Self‐Report and Autonomic Measures

We performed two‐sample *t*‐tests to compare the reported side effects between the taVNS and sham groups. The effects of stimulation on autonomic reactivity and salivary levels were tested using mixed ANOVAs with the within‐subject factors *Time* (pre‐ vs. post‐stimulation) and the between‐subject factor *Stimulation* (taVNS vs. Sham).

#### 3.3.2 Behavioral Performance

##### 3.3.2.1 Recognition Memory

Only participants who could discriminate old vs. new items were included in the analysis (overall Pr index > 0). To calculate the Pr indexes, we considered the correctly identified old items (i.e., hits) and mistakenly identified new items (i.e., false alarms). Old judgments included responses from anchors 4–6. New judgments included responses from anchors 1–3. Six participants were excluded from the memory‐related analysis due to a lower Pr index than 0. Analysis was performed for both the Pr and d‐prime, using linear mixed models, with the factors *Affective Category* (Unpleasant vs. Neutral), *Stimulation* (taVNS vs. Sham) and *Stimulation Phase* (On vs. Off) and their interaction as fixed factors. Participants intercepts were modeled as random effects. Significance was estimated using the R package *lmerTest*, which estimates the degrees of freedom using Satterthwaite approximation.

##### 3.3.2.2 Recognition Memory Based on Confidence Ratings

Memory performance was divided based on the responses of the 6‐point scale to differentiate between familiarity and recollection processes. Recollection‐based memory indexes (Pr and d‐prime) were calculated based on trials answered with anchor 6, whereas Familiarity‐based memory was calculated considering trials answered with anchors 4 and 5. The modulating effects of taVNS on memory recognition based on confidence ratings were evaluated, using linear mixed models with the factors *Affective Category*, *Stimulation*, *Stimulation Phase*, and *Memory* (recollection‐ vs. familiarity‐based) and their interactions. Participants intercepts were modeled as random effects. Significance was estimated using the R package *lmerTest*, which estimates the degrees of freedom using Satterthwaite approximation.

As for Study 1, we used Bayes hypothesis testing analysis (Wagenmakers et al. 2018) to evaluate our hypotheses.

#### 3.3.3 Electrophysiology: Cluster‐Based Permutation Test

##### 3.3.3.1 Encoding

Individual ERP averages were computed for each sensor, *Affective Category* (neutral and unpleasant), *Stimulation* (taVNS and Sham), and *Stimulation phase* (on and off). Four participants were excluded due to bad data quality, leaving a total of 61 participants for the analysis. To assess the interaction effects of stimulation on the processing of negative and neutral images, the *Affective Category* × *Stimulation* interaction as well as the *Affective Category* × *Stimulation* × *Stimulation phase* interactions were submitted to the cluster‐based permutation analysis over posterior sites on an earlier (200–600 ms) and later (600–1200 ms) time window. In addition to the interaction effects, the main effects of *Affective Category* and *Stimulation* were further tested (Supporting Information S4 and S5).

##### 3.3.3.2 Retrieval

Preliminary analysis revealed no interaction effects of *Stimulation phase*, in line with encoding and memory performance findings (critical cluster mass = 1363; largest significant cluster mass = 1278.2). In turn, individual ERP averages were computed for each sensor, *Affective* *Category* (neutral and unpleasant), *Memory* (old and new) and *Stimulation* (taVNS and sham) conditions. In addition to the six participants who were excluded for chance‐level memory recognition, seven participants were excluded due to bad data quality or problems during recording, leaving a total of 52 participants for the analysis. We ran the cluster‐based permutation test over both anterior and posterior sites, in the 400–1000 ms time window, on the three‐way interaction between the *Affective Category* (neutral and unpleasant), *Memory* (old and new) and *Stimulation* (correctly identified old images under taVNS and under Sham stimulation). In addition to the three‐way interaction effects, the main effects of *Memory* and the interaction *Memory* × *Affective Category* effects were further tested (Supporting Information S6 and S7).

For both encoding and retrieval, significant interaction effects were followed by pairwise comparison to unravel the nature of the interaction. In addition, Bayesian hypothesis testing of the significant interaction effects was carried out, using Bayesian ANOVAs on *JASP*. the model with the significant interaction effects was compared to a null model without such interaction, using participants' intercepts as random factors.

### 3.4 Results

#### 3.4.1 Self‐Report and Autonomic Measures

No differences were found in the self‐reported side‐effect ratings between the taVNS and sham groups (all *ps* > 0.41). Results are reported in Table 4.

**TABLE 4:** Mean subjective ratings (standard deviation) for the stimulation side effects (rated from 1, *not at all*, to 7, *very much*) in the active and sham groups (including t‐test comparing both conditions) in Study 2.

	taVNS	sham	*t*‐test
Headache	1.5 (0.8)	1.5 (0.97)	*t*_56.3_ = 0, *p* = 1
Nausea	1.16 (0.57)	1.07 (0.37)	*t*_53_ = 0.74, *p* = 0.46
Dizziness	1.31 (0.78)	1.27 (0.64)	*t*_59_ = 0.25, *p* = 0.8
Neck pain	1.56 (1.05)	1.43 (0.97)	*t*_60_ = 0.50, *p* = 0.61
Neck contraction	1.62 (0.91)	1.73 (1.11)	*t*_56.1_ = −0.42, *p* = 0.67
Stinging sensation	1.97 (1.36)	1.7 (1.18)	*t*_59.7_ = 0.83, *p* = 0.41
Ear irritation	1.5 (1.32)	1.33 (0.99)	*t*_57.4_ = 0.56, *p* = 0.57
Concentration	2.72 (1.71)	2.8 (1.75)	*t*_59.5_ = −0.18, *p* = 0.85
Fluctuation of feelings	1.89 (1.37)	2.2 (1.58)	*t*_57.4_ = −0.82, *p* = 0.41
Unpleasant feelings	2.12 (1.26)	2.1 (1.24)	*t*_60_ = 0.08, *p* = 0.94

##### 3.4.1.1 Autonomic Measures

Mean (and standard deviation) scores of the automatic measures are presented in Table 5. None of the autonomic measures showed significant effects. Heart rate: *Time*, *F*(1, 61) = 1.71, *p =* 0.20, *η*_*p*_^*2*^ = 0.03, *Stimulation*, *F*(1, 29) = 0.26, *p* = 0.61, *η*_p_^2^ = 0.0, *Time* × *Stimulation* interaction, *F*(1, 61) = 0.52, *p* = 0.47. Systolic blood pressure: *Time*, *F*(1, 61) = 0.96, *p* = 0.33, *η*_p_^2^ = 0.01, *Stimulation*, *F*(1, 61) = 0.09, *p* = 0.76, *η*_p_^2^ = 0.0, *Time* × *Stimulation* interaction, *F*(1, 61) = 1.87, *p* = 0.18, *η*_p_^2^ = 0.16. Diastolic blood pressure: *Time*, *F*(1, 61) = 0.29, *p* = 0.86, *η*_*p*_^*2*^ = 0.03, *Stimulation*, *F*(1, 61) = 0.20, *p* = 0.65, *η*_p_^2^ = 0.0, *Time* × *Stimulation* interaction, *F*(1, 29) = 2.85, *p =* 0.09, *η*_p_^2^ = 0.04.

**TABLE 5:** Mean (standard deviation) values of the autonomic measures before and after stimulation in Study 2.

	Time	Heart rate (bpm)	Systolic blood pressure (mmHg)	Diastolic blood pressure (mmHg)
taVNS	Pre	71.61 (10.95)	107.18 (9.37)	75.63 (7.22)
	Post	71.11 (10.6)	106.21 (9.69)	74.5 (6.81)
Sham	Pre	71.1 (11.0)	102.97 (23.37)	75.1 (6.68)
	Post	69.2 (10.71)	108.76 (8.47)	76.5 (6.55)

#### 3.4.2 Behavioral Performance

##### 3.4.2.1 Recognition Memory

To determine whether the factor *Stimulation Phase* (On vs. Off) improved the model fit, we compared a model including *Stimulation Phase* as an interacting factor with *Affective Category* and *Stimulation*, with a model without *Stimulation Phase*. For both Pr and d‐prime indexes, results revealed that the model fit did not improve when *Stimulation Phase* was included; thus, we left it out of the model. Descriptive values are reported in Table 6 (see Section S9 of the Supporting Information).

**TABLE 6:** Mean (standard deviation) of behavioral indices for unpleasant and neutral scenes encoded under sham and taVNS stimulation in Study 2.

	Sham		taVNS	
	Unpleasant	Neutral	Unpleasant	Neutral
Item recognition				
Pr	0.54 (0.18)	0.43 (0.19)	0.57 (0.16)	0.47 (0.17)
d’	1.62 (0.63)	1.27 (0.68)	1.79 (0.63)	1.41 (0.67)
Recognition memory based on confidence ratings				
Familiarity‐based PR	0.08 (0.14)	0.12 (0.13)	0.05 (0.15)	0.10 (0.14)
Recollection‐based PR	0.42 (0.18)	0.26 (0.17)	0.49 (0.19)	0.32 (0.21)
Familiarity‐based d’	0.28 (0.49)	0.37 (0.47)	0.16 (0.59)	0.36 (0.54)
Recollection‐based d’	1.96 (0.86)	1.59 (0.96)	2.24 (0.65)	1.97 (0.77)

For Pr, a linear mixed model revealed an effect of *Affective Category*, *t*(166) = 4.74, *p* < 0.001, but no significant main effects of *Stimulation*, *t*(70.3) = −1.03, *p* = 0.30, or interaction effects, *t*(166) = 0.53, *p* = 0.60, were observed. This was confirmed by Bayesian hypothesis testing, as denoted by decisive evidence for an effect of *Affective Category*, *BF*_10_ > 100, but a lack of evidence for an effect of *Stimulation*, BF_10_ = 0.43, or interaction, BF_10_ = 0.20.

For d‐prime, an effect of *Affective Category* emerged, *t*(166) = 4.67, *p* < 0.001, but no significant effect of *Stimulation*, *t*(70.13) = −0.88, *p* = 0.37, or interaction effects, *t*(166) = −0.22, *p* = 0.82. These results were paralleled by the Bayesian hypothesis testing analysis, as indicated by decisive evidence for an effect of *Affective Category*, BF_10_ > 100, but a lack of evidence for an effect of *Stimulation*, BF_10_ = 0.55, or interaction, BF_10_ = 0.20.

##### 3.4.2.2 Recognition Memory Based on Confidence Ratings

**FIGURE 5:**
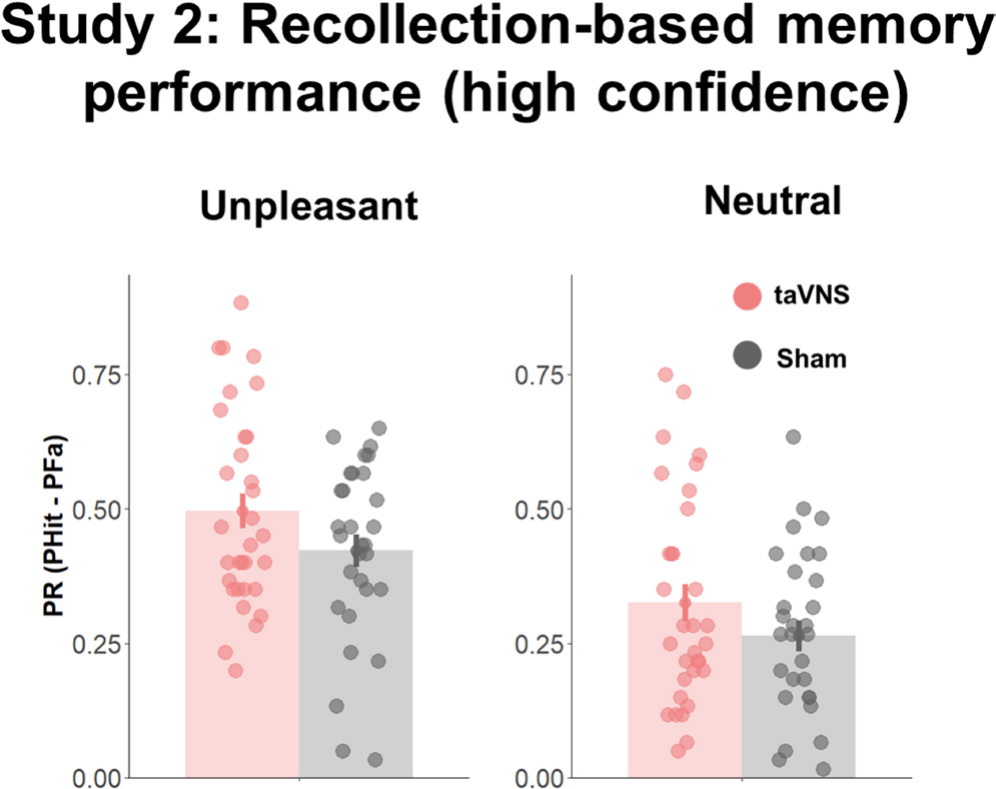
Memory performance for recollection‐related (highest confidence) judgments. Depiction of behavioral performance for unpleasant and neutral images of Study 2. Error bars represent standard error. Bar plots represent mean values. Dots indicate individual scores.

When memory performance was split into recollection‐ and familiarity‐based responses for the Pr index, an effect of *Memory* was observed, *t*(428) = 7.98, *p* < 0.001, indicating higher recollection‐ than familiarity‐based memory performance, but no *Affective Category*, *t*(428) = −1.82, *p* = 0.06, or *Stimulation* effects were found, *t*(256) = 0.42, *p* = 0.67. Although no *Affective Category* × *Stimulation* was observed, *t*(428) = 0.33, *p* = 0.73, the *Affective Category* × *Memory* interaction was significant, *t*(428) = 5.66, *p* < 0.001, and the *Memory* × *Stimulation* interaction approached significance, *t*(232) = −1.89, *p* = 0.059. No triple *Affective Category* × *Stimulation* × *Memory* interaction was found, *t*(428) = −0.49, *p* = 0.66 (Figure 5). Following up on the interaction effects approaching significance, we could observe that the taVNS and sham group reported similar familiarity‐based memory, *t*(120) = −0.78, *p* = 0.43, but the taVNS group showed a recollection‐based memory advantage, *t*(120) = 2.3, *p* = 0.01. Further post hoc analysis revealed that both neutral, *t*(428) = −9.20, *p* < 0.001, and unpleasant images, *t*(428) = −19.73, *p* < 0.001, showed a recollection‐based memory advantage. Bayes hypothesis testing revealed decisive evidence for *Memory*, BF_10_ > 100, and *Affective Category*, BF_10_ > 100, but not for *Stimulation*, BF_10_ = 0.31. Although moderate evidence for a lack of an *Affective Category* × *Stimulation* effect was observed, BF_10_ = 0.13, decisive evidence was found for the *Affective Category* × *Memory* interaction, BF_10_ > 100, and moderate evidence for the *Memory* × *Stimulation* interaction, BF_10_ = 6.63. Moreover, no evidence for the triple interaction was found, BF_10_ = 0.18.

For the d‐prime index, we observed an effect of *Memory*, *t*(428) = 15.76, *p* < 0.001, indicating heightened recollection than familiarity‐based memory performance, and an effect of *Affective Category*, *t*(428) = 3.71, *p* < 0.001, suggesting better memory performance for unpleasant compared to neutral images, but no effects of *Stimulation* were observed, *t*(157) = 0.04, *p* = 0.96. In the absence of an *Affective Category* × *Stimulation* interaction, *t*(428) = 0.80, *p* = 0.42, an *Affective Category* × *Memory*, *t*(428) = 3.31, *p* = 0.001, as well as *Memory* × *Stimulation* interaction was found, *t*(428) = −2.65, *p* = 0.008. The *Affective Category* × *Stimulation* × *Memory* interaction was not significant, *t*(428) = −0.43, *p* = 0.96.

Follow‐up analysis on the *Memory* × *Stimulation* interaction revealed that although the taVNS and sham group showed comparable familiarity‐based memory, *t*(150) = −0.59, *p* = 0.55, taVNS increased the recollection‐based memory performance compared to sham stimulation, *t*(150) = 2.60, *p* = 0.01. Following up on the *Affective Category* × *Memory* interaction, we could also observe that while familiarity‐ and recollection‐based memory performance was comparable for neutral images, *t*(428) = −0.026, *p* = 0.97, a recollection advantage was observed for unpleasant images, *t*(428) = −10.62, *p* < 0.001.

Bayes hypothesis testing further revealed moderate evidence against an effect of *Affective Category*, BF_10_ = 0.21, and anecdotal evidence against *Stimulation* effects, BF_10_ = 0.75, but decisive evidence for *Memory* effects, BF_10_ > 100. Moreover, moderate evidence for a lack of an *Affective Category* × *Stimulation* interaction was found, BF_10_ = 0.15, but decisive evidence for an *Affective Category* × *Memory* interaction, BF_10_ > 100, and strong evidence for a *Memory* × *Stimulation* interaction, BF_10_ = 16.98. No evidence for a triple interaction was observed, BF_10_ = 0.21.

#### 3.4.3 Electrophysiological Analyses: Permutation Test

##### 3.4.3.1 Encoding

Cluster‐based permutation tests on the main effects of *Affective Category* and *Stimulation* are reported in Sections S4 and S5 of the Supporting Information.

**FIGURE 6:**
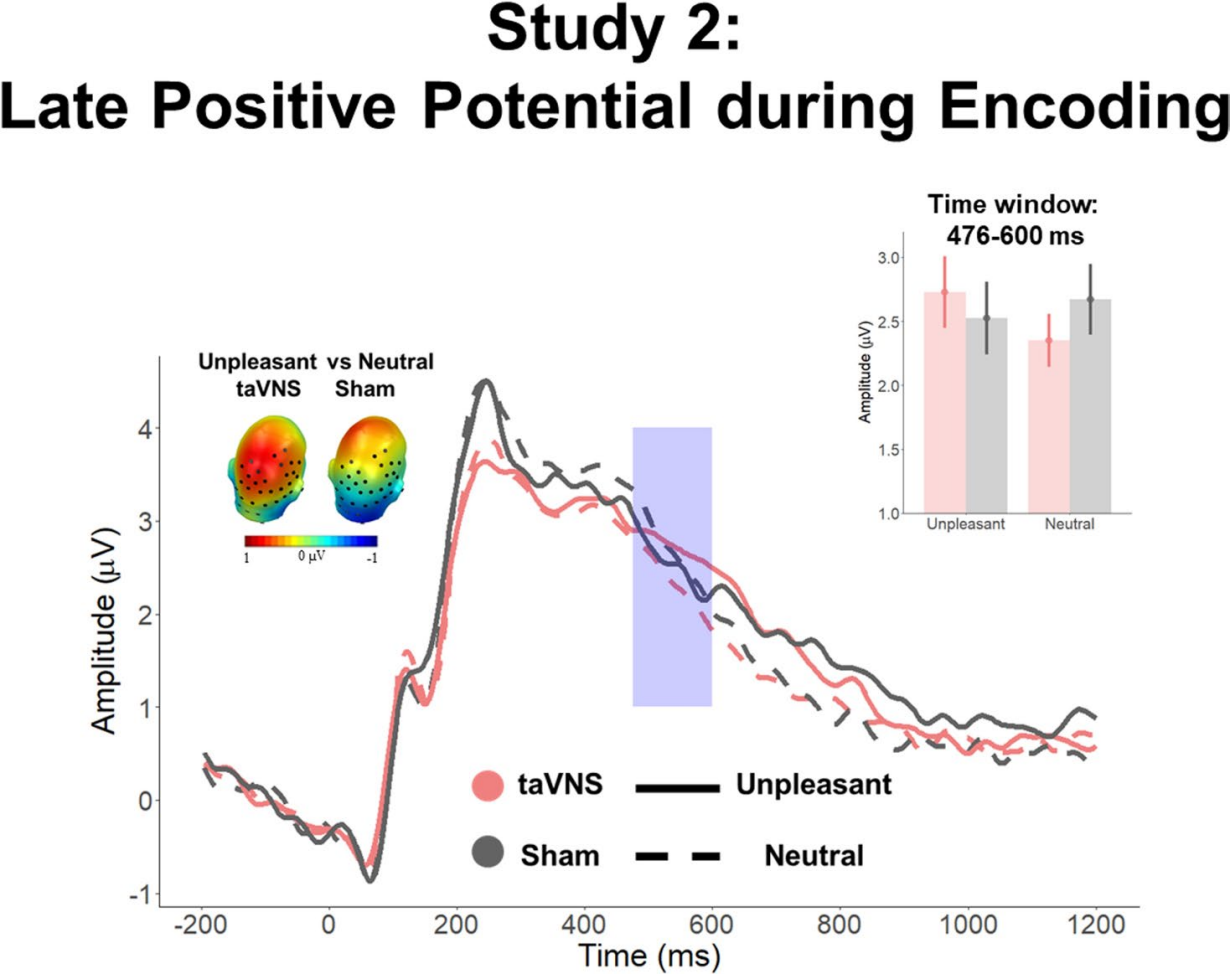
ERP results during encoding in Study 2. ERP‐averaged waveforms across the significant sensor clusters. The blue box indicates the significant time window. Upper right inset: Mean averaged ERPs during the significant time window and sensor clusters, showing a larger emotion discrimination during taVNS than sham condition. Upper left inset: Scalp topographies showing emotional differences during for taVNS and sham groups, separately.

For the *Affective Category* × *Stimulation* interaction in the early time window (200–600 ms), one cluster over a posterior region surpassed the cluster mass of 1728 (Cluster 1: mass = 1980.3, time window = 476–600 ms, sensors = 60, 61, 62, 65, 66, 67, 69, 70, 71, 72, 74, 75, 76, 77, 79, 81, 82, 83, 84, 86, 87, 88, 90, 91, 92, 93, 96, 97, 98, 101, 102, 103, and 108). Analysis on Cluster 1 showed no main effect of *Affective Category*, *F*(1, 59) = 1.17, *p* = 0.29, *η*_p_^2^ = 0.02 or *Stimulation*, *F*(1, 59) = 0.027, *p* = 0.87, *η*_p_^2^ = 0.00, but a significant interaction *Affective Category* × *Stimulation* emerged, *F*(1, 59) = 5.63, *p* = 0.02, *η*_p_^2^ = 0.09 (BF_10_ = 2.79). Subsequent *t*‐test analyses revealed significant differences in the LPP amplitude between neutral and unpleasant images in the taVNS group, *t*(30) = −2.21, *p* = 0.035, but not in the sham group, *t*(29) = 1.05, *p* = 0.3. However, no differences between the taVNS and sham group were found for unpleasant, *t*(58.9) = −0.51, *p* = 0.61, or neutral images, *t*(54.3) = −0.93, *p* = 0.36 (Figure 6).

For the interaction *Affective Category* × *Stimulation* × *Stimulation Phase*, no cluster surpassed the critical cluster mass of 1728.

In the late time window (600–1200 ms), two clusters over the posterior region were larger than the critical cluster mass of 765 (Cluster 1: mass = 5229.8, time window = 712–1056 ms; sensors = 55, 75, 76, 78, 79, 82, 83, 84, 85, 86, 87, 88,89, 90, 91, 92, 93, 91, 92, 93, 94, 95, 96, 97, 98, 99, 100, 101, 102, 103, 107, 108, and 113; Cluster 2: mass = 3139.9, time window = 1132–1200 ms: 68, 69, 73, 74, 75, 76, 77, 81, 82, 83, 84, 85, 86, 88, 89, 90, 91, 92, 93, 94, 95, 96, 97, 98, 99, 100,101, 102, 103, 107, 108, and 113). Analysis of Cluster 1 showed a main effect of *Affective Category*, *F*(1, 59) = 4.93, *p* = 0.03, *η*_*p*_^*2*^ = 0.07, but no effect of *Stimulation*, *F*(1, 59) = 0.001, *p* = 0.97, *η*_p_^2^ = 0.00. However, a significant interaction between *Affective Category* × *Stimulation* was found, *F*(1, 59) = 8.16, *p* = 0.006, *η*_p_^2^ = 0.12 (BF_10_ = 7.29). Post hoc comparisons revealed larger amplitudes for unpleasant compared to neutral images in the sham group, *t*(29) = −3.16, *p* = 0.003, but not in the taVNS group, *t*(30) = 0.57, *p* = 0.60. However, no differences between the taVNS and sham groups were found for unpleasant, *t*(58.6) = −1.45, *p* = 0.15, or neutral images, *t*(52.7) = −1.16, *p* = 0.25. Analysis of Cluster 2 revealed no main effects of *Affective Category* or *Stimulation* (Fs < 1), but an interaction between *Affective Category and Stimulation*, *F*(1, 59) = 11.99, *p* = 0.001, *η*_p_^2^ = 0.17 (BF_10_ = 6.79). Subsequent analysis indicated that participants in the sham group showed larger amplitudes for unpleasant than neutral images, *t*(29) = −3.11, *p* = 0.004, whereas no differences were found for participants in the taVNS group, *t*(30) = 1.57, *p* = 0.12. Participants in the sham, compared to the taVNS group, also showed larger amplitudes for unpleasant images, *t*(59) = −2.39, *p* = 0.02, but no differences were found for neutral images, *t*(51.8) = 1.14, *p* = 0.26.

For the interaction *Affective Category* × *Stimulation* × *Stimulation Phase*, no cluster surpassed the critical cluster mass of 833.5.

##### 3.4.3.2 Retrieval

Cluster‐based permutation tests on the main effects of *Memory* and interaction effects of *Memory* × *Affective Category* are reported in Sections S6 and S7 of the Supporting Information.

**FIGURE 7:**
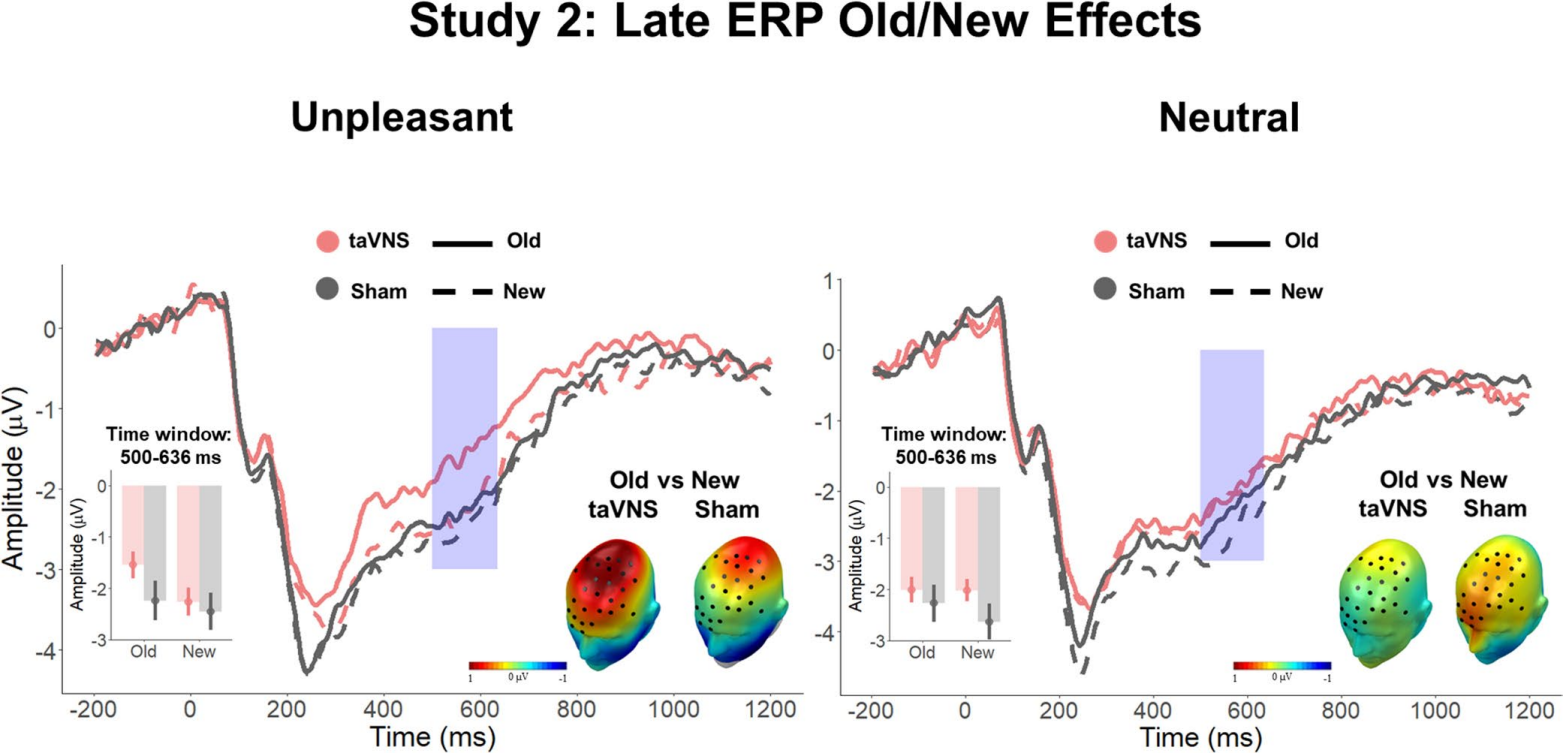
ERP results during retrieval in Study 2. ERP‐averaged waveforms across the significant sensor cluster. Left for unpleasant images and right for neutral images. The blue box indicates the significant time window. Left inset: Mean averaged ERPs during the significant time window (500–636 ms) and sensor cluster, showing larger ERP Old/New effects for unpleasant images in the taVNS group compared to the sham group. Right inset: Topographical Old/New differences for the taVNS and sham groups, separately.

To test for the interaction effects of affective category and stimulation on memory retrieval, we submitted the three‐way interaction *Affective Category* (unpleasant vs. neutral) × *Stimulation* (taVNS group vs. sham group) × *Memory* (old vs. new) to a cluster‐based permutation test for the 400–1000 ms time window over both anterior and posterior electrode regions. One cluster with a mass of 1658.5 surpasses the critical mass of 1252.5 (sensors: 2, 3, 4, 5, 6, 7, 9, 10, 11, 12, 13, 15, 16, 18, 19, 20, 24, 28, 29, 30, 31, 34, 36, 37, 105, 106, 111, and 129; time window: 500–636 ms). Analysis on Cluster 1 revealed no main effects of *Affective Category*, *F*(1, 50) = 1.95, *p* = 0.16, *η*_p_^2^ = 0.04, or *Stimulation*, *F*(1, 50) = 1.0, *p* = 0.32, *η*_p_^2^ = 0.02; however, a main effect of *Memory* emerged, *F*(1, 50) = 21.57, *p* < 0.001, *η*_p_^2^ = 0.31. Neither a *Stimulation* × *Affective Category* nor a *Stimulation* × *Memory* interaction reached significance (Fs < 1), but the interaction *Affective Category* × *Memory* approached significance, *F*(1, 50) = 3.67, *p* = 0.061, *η*_p_^2^ = 0.068. Most importantly, the three‐way interaction was significant, *F*(1, 50) = 8.73, *p* = 0.005, *η*_p_^2^ = 0.15 (BF_10_ = 9.80). Subsequent analysis revealed larger ERP Old/New differences for unpleasant pictures in the taVNS, *t*(23) = −4.47, *p* < 0.001, but not in the sham group, *t*(27) = −1.56, *p* = 0.13. For neutral images, participants in the sham group showed significant differences between old and new images, *t*(27) = −2.74, *p* = 0.011. Such differences were not observed in the taVNS group, *t*(23) = −0.07, *p* = 0.94; (Figure 7).

### 3.5 Discussion

Based on the findings from our previous study (Ventura‐Bort et al. 2021) and Study 1, in Study 2 we investigated whether concurrent stimulation is critical for the enhancing effects of taVNS on emotional processing and retrieval. We therefore synchronized the on and off‐cycles of the stimulation with the encoding of unpleasant and neutral images to differentiate between stimuli encoded during stimulation or not. During encoding, we observed that emotional images evoked larger LPP amplitudes than neutral ones (Supporting Infotmation S4), in line with previous findings (Ribes‐Guardiola et al. 2023; Schupp et al. 2000). Moreover, the LPP amplitudes during the early time window (476–600 ms) interacted with the stimulation group. More concretely, unpleasant images evoked larger amplitudes than neutral images in the taVNS compared to the sham group. However, no effects of the stimulation phase were found. These results are consistent with Study 1 and with our previous findings (Ventura‐Bort et al. 2021) and support the evidence for a modulatory effect of taVNS on emotional processing.

During retrieval, we observed that old images elicited larger amplitudes than correctly identified new ones (Supporting Infotmation S6), particularly for emotionally salient stimuli (Supporting Infotmation S7), replicating previous research (Weymar et al. 2009; Weymar and Hamm 2013). More interestingly, a triple interaction revealed larger late Old/New differences for unpleasant images in the taVNS compared to the sham group. These ERP findings also conceptually replicate our previous results (Study 1 and Ventura‐Bort et al. 2021) indicating a recollective advantage for unpleasant material encoded under taVNS, which is not influenced by the stimulation cycle during active taVNS.

On the behavioral level, we observed an enhancement in recollection memory in the taVNS compared to the sham group, irrespective of affective category and stimulation cycle. These results suggest that the effects of taVNS on memory may not be dependent specifically on the synchronization between task events and stimulation periods (see general discussion for other factors that may need to be considered).

## 4 General Discussion

In the current studies, we aimed to replicate and extend our previous findings on the role of the VN in encoding and retrieval of emotionally relevant information by investigating whether interval stimulation (in contrast to continuous stimulation used in our previous study; Ventura‐Bort et al. 2021) modulates the taVNS enhancing effects on emotional processing and memory. For brain potentials, we could conceptually replicate our previous results. In both Study 1 and Study 2, we observed that taVNS compared to sham stimulation increased the processing of unpleasant relative to neutral stimuli, as reflected by larger amplitudes in an earlier time window of the LPP (Study 1: 416–548 ms; Study 2: 476–600 ms). Furthermore, unpleasant images encoded under taVNS produced larger ERP Old/New differences compared to the sham stimulation during the recognition task (1 week later) in a time window related to recollection‐based retrieval (Study 1: 672–776 ms; Study 2: 500–636 ms). At the behavioral level, although no stimulation effects were observed for memory performance in Study 1, we observed in Study 2 (when the stimulus presentation was synchronized to the stimulation cycle) that participants showed better recollection‐based retrieval for both unpleasant and neutral images compared to sham. This enhancement in memory, however, occurred irrespective of whether the stimuli were encoded during the on or off cycle of the stimulation. Altogether, these findings suggest significant modulatory effects of taVNS on emotional encoding and retrieval and strengthen the existing evidence about the contribution of the VN to these processes. Our results also show that changes in time‐dependent stimulation parameters (either continuous or 30 s on/off interval stimulation) may play a less critical role in modulating these effects.

Existing models propose that the VN and the LC are key nodes for emotional processing and episodic memory (Mather et al. 2016; McGaugh 2004; McIntyre et al. 2012). The VN participates in the transmission of information about peripherally released stress hormones from the adrenal gland to the brain. In the context of experiencing an emotional event, vagal afferences promote central noradrenergic release in the LC by innervating the NTS. Consequently, the secretion of noradrenaline from the LC varicosities influences the catecholaminergic release in regions important for emotional processing and memory formation, such as the AMY, HC, and frontal cortex (Mather et al. 2016; McIntyre et al. 2012). Supporting the view of VN on LC activity, animal research found that VNS increases LC firing rates (Dorr and Debonnel 2006) and hippocampal activity (Raedt et al. 2011; Roosevelt et al. 2006) in rats. Extending these findings to humans, recent functional imaging research has shown that taVNS compared to sham stimulation increases the activity of the LC (Frangos et al. 2015; Müller et al. 2022; Teckentrup et al. 2021; Yakunina et al. 2017). In line with our previous ERP results (Ventura‐Bort et al. 2021) in both Study 1 and Study 2, we found that taVNS modulates the encoding of emotional and neutral information. More concretely, we did observe a preferred processing of emotional vs. neutral material during taVNS compared to sham stimulation, as demonstrated by larger ERP differences in an earlier time window of the LPP, suggesting that taVNS may facilitate the detection of salient signals. These results are consistent with the *glutamate amplifies noradrenergic effects* (GANE) model (Mather et al. 2016) that assumes that the activation of the LC may enhance the processing of relevant information at the expense of non‐relevant material (for potential link between LC and LPP, see Hajcak and Foti 2020).

In relation to memory formation, the modulation of the AMY and HC via noradrenergic release has been shown to specifically impact recollection‐based episodic memory and its surrogates, such as the late ERP Old/New effect (Weymar et al. 2010; Wirkner et al. 2013). In both studies, the interaction effects of taVNS on emotional episodic memory revealed larger Old/New differences (i.e., less negative deflections for correctly recognized unpleasant images compared to new ones). As for encoding, the retrieval data also replicated conceptually our previous findings (Ventura‐Bort et al. 2021), strengthening the notion that the VN may also exert a significant influence on the recollection‐related neural circuitry. It should be pointed out that in both Study 1 and Study 2, the interaction effects observed in the ERP Old/New effects were central‐frontally located, whereas in our previous study, they showed a more posterior distribution. Although recollection memory has been associated with hippocampal activity (Dolcos et al. 2005), parietal and frontal areas, including the orbital and superior frontal cortex, also contribute to recollection processes (Ventura‐Bort et al. 2024; Ventura‐Bort, Wendt, et al. 2020), as part of the so‐called recollection network (Rugg and Vilberg 2013). The fact that the interaction effects of stimulation were more frontally and centrally located may be interpreted as a stronger involvement of frontal regions during the recollective experience. Another reason for the topographical differences between studies may be related to the mnemonic processes involved. Previous studies have associated frontal ERP Old/New effects during the initial 300–500 ms after stimulus onset with familiarity‐based retrieval (Rugg and Curran 2007). It could, thus, be that the current results reflect both familiarity‐ and recollection‐based processes. Considering that the frontal, familiarity‐related Old/New effects have typically been identified in earlier time windows than the ones here reported, which coincide with the late, recollection‐related ERP Old/New effects, and the fact that our taVNS effects increased particularly recollection‐based performance (in Study 2), our ERP results may indicate that taVNS increased recollection rather than familiarity processes.

It should also be noted that, unlike our previous study, the recollection enhancement effects of taVNS for emotional information was found for the ERPs but not in the memory performance. In Study 2, but not in Study 1, we found a general recollection advantage in memory performance, irrespective of emotionality and stimulation cycle (e.g., Giraudier et al. 2020; Jacobs et al. 2015; but see Mertens et al. 2022). There might be different factors contributing to the divergent effects of taVNS on the behavioral level between studies and to the discrepancy between behavioral and the ERP results that warrant future investigation. One potential factor contributing to study differences is the sample size. Study 1 had a considerably smaller sample size compared to Study 2 (see also Giraudier et al. 2020; Ventura‐Bort et al. 2021). Thus, the lack of behavioral differences in Study 1 may be due to the lack of power^4^. Another influencing source of variability may be associated with the material that was used in the present studies. Although the current pictures were selected from standardized datasets and matched for arousal, it could also be that the experienced arousal was not equal across participants, reducing the interaction effects of taVNS and emotion (see also Giraudier et al. 2020). Regarding the discrepancy between behavioral and ERP findings, the fact that we could not observe the expected recollection‐based advantage for unpleasant images encoded under taVNS in memory performance may be related to the precision of the recollection index used. In our studies, recollection‐based memory performance was assessed based on the highest reported confidence ratings. Although higher confidence ratings have been associated with recollection processes (e.g., Rimmele et al. 2011; Perfect et al. 1996), other measures of recollection (e.g., recall test, choose‐from‐array test) that more accurately inform about the amount of details retrieved may have provided a better approximation of the recollection‐based taVNS advantage for emotional information at the behavioral level.

Despite the differences between studies at a behavioral level, we were able to conceptually replicate our previous finding on taVNS enhancing effects in brain potentials by also showing that the modulating effects of taVNS on electrophysiological indexes of processing and memory retrieval for emotional contents may be independent of the stimulation phase in which they are encoded. These results may indicate that the effects of taVNS on emotional processing and memory are due to the modulation of tonic rather than phasic neurobiological responses. It has been proposed that the phasic activity of the LC is directly dependent on its tonic levels. Specifically, it has been shown that at lower and higher tonic levels, phasic responses are low, whereas at intermediate tonic levels, phasic responses are high (Aston‐Jones and Cohen 2005). This mechanism has also been suggested to be reflected at an electrophysiological level (Lu et al. 2023; Murphy et al. 2011; Nieuwenhuis et al. 2005). In relation to our findings, it could thus be that taVNS modulates the tonic levels of the LC, optimizing phasic LC responses that favor both the encoding (Mather and Sutherland 2011) and memory storage (Mather et al. 2016; McGaugh 2004) of high‐priority (emotionally salient) information. Similar effects have been found in stress studies, showing that increased tonic arousal levels favor processing and memory for emotional information (Weymar et al. 2012; Wirkner et al. 2013).

The current findings also highlight the potential of taVNS as a non‐invasive technique for modulating mnemonic processes in non‐clinical and clinical populations. The growing evidence that taVNS can improve various cognitive processes in healthy participants (Borges et al. 2021; De Smet et al. 2021; Sommer et al. 2023) has led to the suggestion that non‐invasive stimulation of the VN may be a promising tool to reduce cognitive decline in aging (Naparstek et al. 2023) and cognitive disorders (Broncel et al. 2020; Colzato and Beste 2020). In support, a recent clinical trial demonstrated that taVNS may help improve cognitive functions in patient with mild cognitive impairments (Wang et al. 2023). Our results suggest that the beneficial effects of taVNS may extend to episodic memory processes, opening new venues to improve the mnemonic impairments associated with clinical conditions such as dementia or general memory decline in aging.

Although the conceptual replication of the effects of taVNS serves to strengthen our previous findings, there are some aspects that need to be considered in future research on the impact of taVNS on emotional episodic memory. The sample of the current studies consisted of young adults, mostly women. It has been shown that hormonal fluctuations associated with the menstrual cycle modulate the retrieval of emotional memories (Bayer et al. 2014). Unfortunately, we did not collect information about the menstrual cycle to investigate its impact on emotional episodic memory. Future studies examining whether the effects of taVNS interact with the menstrual cycle to modulate emotional episodic memory may thus help to advance our knowledge about the mechanism of action and efficacy of taVNS.

In conclusion, our results indicate that taVNS may enhance the initial processing and subsequent retrieval of emotionally relevant material, pointing to a prominent role of the VN in these processes. These findings further invite to consider taVNS as a promising tool to improve memory processes in clinical and non‐clinical conditions associated with memory decline.

## Author Contributions

**Carlos Ventura‐Bort:** conceptualization, data curation, formal analysis, investigation, methodology, project administration, software, supervision, validation, visualization, writing – original draft, writing – review and editing. **Manon Giraudier:** investigation, project administration, writing – review and editing. **Mathias Weymar:** conceptualization, funding acquisition, project administration, resources, writing – review and editing.

## Conflicts of Interest

The authors declare no conflicts of interest.

## Supporting information


Data S1.

## Data Availability

Data will be available upon request.

## Endnotes

^1^
It should be noted that although the behavioral parameters (Pr and d‐prime) were extracted using the same procedure as in Ventura‐Bort et al. (2021), the analysis strategy changed. In Ventura‐Bort et al. (2021), we used analysis of variance (ANOVAs), whereas in the current study linear mixed models (lmms) were employed.

^2^
For those cases in which false alarms were 0, the value was substituted by 1/(total of trials*2). Total of trials = 240.

^3^
In contrast to Study 1 in which a within‐subject design was used, Study 2 consisted of a mixed design for methodological reasons. In study 2, we wanted to keep the same number of scenes per conditions (*n* = 30) as in Study 1 and our previous study (Ventura‐Bort et al. 2021), without having to extend the procedure excessively in any of the sessions. In a within‐subject design, participants would have been presented with 480 images (instead of 240 scenes) during retrieval, doubling the length of the retrieval session. Although different designs may make it difficult to directly compare results between studies, it also ensures that results, if replicated, do not depend on the characteristics of the design per se, further strengthening the conclusions that could be drawn about the effects of taVNS.

^4^
A posteriori sample size calculation, using G*Power 3.1.9.7 for repeated‐measures ANOVA based on the effect sizes of or previous study (f = 0.19, 1‐β = 0.80, alpha‐level = 0.05, 2 groups, 2 measurements) revealed that the optimal sample size for detecting small significant effects, would be 58 participants. Although this sample size is reached in Study 2, the sample size in Study 1 is considerably smaller.
