# Supplementary material for: Transcutaneous Auricular Vagus Nerve Stimulation Enhances Emotional Processing and Long‐Term Recognition Memory: Electrophysiological Evidence Across Two Studies
Source: Psychophysiology. 2025 Mar 11;62(3):e70034. doi: 10.1111/psyp.70034 (PMC11894791; doi:10.1111/psyp.70034)
Supplement: Supplementary file 1 — Data S1. [file PSYP-62-e70034-s001.docx]

**Supplementary Material**

S1. Cluster-based permutation tests revealing *Affective Category* effects during encoding (Study 1).

S2. Cluster-based permutation tests revealing *Stimulation* effects during encoding (Study 1).

S3. Other electrode clusters revealing *Affective Category x Stimulation* interaction effects in the 200-600 ms time window during encoding (Study 1).

S4. Cluster-based permutation tests revealing *Affective Category* effects during encoding (Study 2).

S5. Cluster-based permutation tests revealing *Stimulation* effects during encoding (Study 2)

S6. Cluster-based permutation tests revealing *Memory* effects during retrieval (Study 2).

S7. Cluster-based permutation tests revealing *Memory x Affective Category* effects during retrieval (Study 2).

S8. Cluster-based permutation tests revealing *Affective Category* effects during encoding for the sham condition in the early time window of the LPP (Study 1 and Study 2).

S9. Visual depiction of the memory performance, overall and split by familiarity and recollection.

**S1. Cluster-based permutation tests revealing *Affective Category* effects during encoding (Study 1).**

In the early time window (200 - 600 ms), two clusters over the posterior region surpassed the cluster mass of 884.5 (Cluster 1: mass = 4360.1, time window = 444 - 588 ms, sensors 42, 47, 51, 52, 53, 54, 55, 59, 60, 61, 62, 65, 66, 67, 71, 72, 76, 77, 78, 79, 84, 85, 86, 87, and 91; Cluster 2: mass = 986.7 , time window = 484 - 600 ms, sensors = 99, 100, 101, 107, 108, and 113). Figure S1 depicts the mean amplitudes for each condition for the significant clusters and time windows.

Analysis on Cluster 1 revealed a main effect of *Affective Category,* F(1,25) = 14.79, p < .001, *η*_p_^2^ = 0.37, a main effect of *Stimulation,* F(1,25) = 10.09, p = .004, *η*_p_^2^ = 0.28, but no *Affective Category x Stimulation* interaction*,* F(1,25) = 3.39, p = .07, *η*_p_^2^ = 0.12. Despite the lack of a significant interaction, we conducted t-tests to explore the differences between unpleasant and neutral images for the taVNS and sham condition, separately. Results revealed significant differences between unpleasant and neutral scenes encoded under taVNS, t(26) = -3.54, p =.001, but not under sham, t(26) = -1.04, p = .31.

For Cluster 2, a main effect of *Affective Category* was observed, F(1,25) =12.51, p = .002, *η*_p_^2^ = 0.33. Although no *Stimulation* effects emerged, F(1,25) = 0.14, p = .71, *η*_p_^2^ = 0.005, an interaction between *Affective Category* and *Stimulation* was found, F(1,25) = 7.35, p = .012, *η*_p_^2^ = 0.23. Subsequent t-tests revealed significant differences between unpleasant and neutral scenes encoded under taVNS, t(26) = -3.54, p =.001, but not under sham, t(26) = -0.29, p = .77.

In the late time window (600 - 1200 ms), two posterior clusters surpassed the cluster mass of 649.5 (Cluster 1: mass = 21746, time window = 600 - 1012 ms, sensors = 37, 42, 47, 51, 52, 53, 54, 55, 58, 59, 60, 61, 62, 65, 66, 67, 69, 70, 61, 72, 73, 74, 75, 76, 77, 78, 79, 81, 82, 83, 84, 85, 86, 87, 88, 90, 91, 92, 93, 96, 97, 98, 102, 103; Cluster 2: mass = 767.2 , time window = 600 - 788 ms, sensors =107 and 113).

For Cluster 1, results revealed a main effect of *Affective Category,* F(1,25) = 19.01, p < .001, *η*_p_^2^ = 0.43, but no *Stimulation,* F(1,25) = 2.38, p = .13, *η*_p_^2^ = 0.08, or interaction effects*, Affective Category x Stimulation:* F(1,25) =1.02, p = .32, *η*_p_^2^ = 0.04. Although no significant interaction was observed, we conducted t-tests to explore the differences between unpleasant and neutral images for the taVNS and sham condition, separately. Results revealed significant differences between unpleasant and neutral images encoded under both taVNS, t(26) = -3.91, p <.001, and sham stimulation, t(26) = -2.34, p = .027. For Cluster 2, a main effect of *Affective Category* was found*,* F(1,25) = 9.93, p = .004, *η*_p_^2^ = 0.28, but no effect of *Stimulation,* F(1,25) = 0.18, p = .67, *η*_p_^2^ = 0.007, or interaction of *Affective Category and Stimulation,* F(1,25) =0.83, p = .37, *η*_p_^2^ = 0.03. Subsequent t-tests revealed significant differences between unpleasant and neutral images encoded under both taVNS, t(26) = 2.67, p = .013, and sham stimulation, t(26) = 2.38, p = .025.

Altogether, the expected larger amplitudes for emotional compared to neutral scenes were observed in both the earlier LPP (Cluster 1, 444 -588 ms and Cluster 2: = 484 – 600 ms) and later LPP time window (Clusters 1, 600 - 1012 ms). However, for the early time window of the LPP, effects were especially pronounced in the taVNS condition (see section S8 for
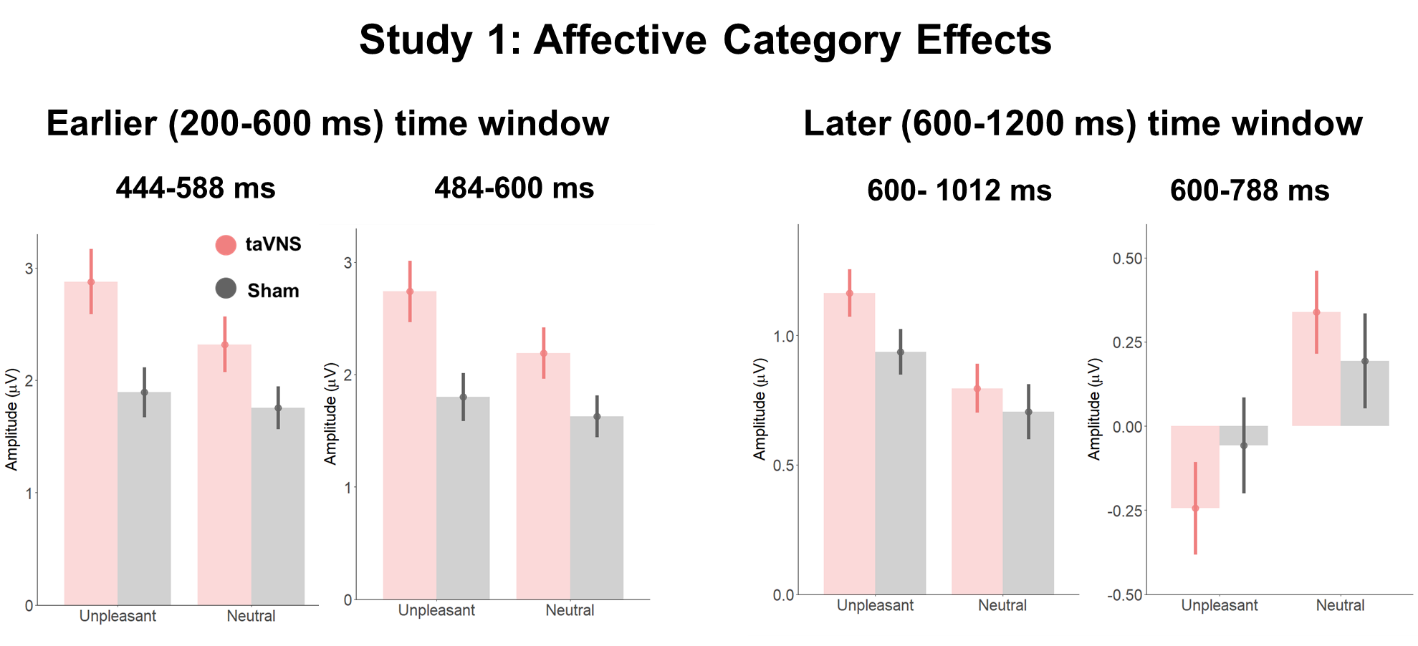
follow-up analysis on the sham condition).

Figure S1. Mean averaged ERPs during the significant time window and sensor clusters, for the earlier (left) and later (right) time windows of the *Affective Category* effects.

**S2. Cluster-based permutation tests revealing *Stimulation* effects during encoding (Study 1).**

In the early time window (200 - 600 ms), one cluster over posterior electrodes surpassed the cluster mass of 891.5 (Cluster 1: mass = 26724.2, time window = 200 - 600 ms, sensors 50, 51, 52, 53, 54, 55, 56, 57, 58, 59, 60, 61, 62, 63, 64, 65, 66, 67, 68, 69, 70, 71, 72, 73, 74, 75, 76, 77, 78, 79, 81, 82, 83, 84, 85, 86, 87, 88, 89, 90, 91, 92, 93, 94, 95, 96, 98, 102, 103, and 108).

Analysis on Cluster 1 revealed a main effect of *Affective Category,* F(1,25) = 12.09, p = .002, *η*_p_^2^ = 0.32, a main effect of *Stimulation,* F(1,25) =7.19, p = .013, *η*_p_^2^ = 0.22, but no interaction *Affective Category x Stimulation,* F(1,25) =3.18, p = .086, *η*_p_^2^ = 0.11. Despite the lack of a significant interaction, we conducted t-tests to explore the differences between conditions for neutral and unpleasant images, separately. Subsequent analyses revealed significant differences between sham and taVNS conditions for unpleasant scenes, t(26) = -3.29, p = .002, but not for neutral ones, t(26) = -1.36, , p = .19.

In the late time window (600 - 1200 ms), three posterior electrode clusters surpassed the cluster mass of 661.5 (Cluster 1: mass = 3704.2, time window = 600-704 ms, sensors = 53, 54, 58, 59, 60, 61, 62, 63, 64, 65, 66, 67, 68, 69, 70, 71, 72, 73, 74, 75, 76, 77, 78, 79, 81, 82, 83, 84, 85, 86, 88, 89, 90, 91, 94, and 95; Cluster 2: mass = 1494.8 , time window = 984 – 1200 ms, sensors = 79, 86, 87, 93, 98, 101, 102, 103, and 108, Cluster 3: mass = 702 , time window = 972 – 1060 ms, sensors = 984).

For Cluster 1, results revealed a main effect of *Affective Category,* F(1,25) =9.22, p < .006, *η*_p_^2^ = 0.27, a main effect of *Stimulation,* F(1,25) = 5.94, p = .022, *η*_p_^2^ = 0.19, but no interaction *Affective Category x Stimulation,* F(1,25) = 1.48, p = .24, *η*_p_^2^ = 0.06. Although the interaction was not significant, we conducted t-tests to explore the differences between conditions for neutral and unpleasant images, separately. Results revealed significant differences between sham and taVNS conditions for unpleasant scenes, t(26) = -2.71, p = .012, but not for neutral ones, t(26) = -1.50, p = .14. For Cluster 2, and Cluster 3, no significant effect of *Stimulation* was observed (Fs< 1).

**S3. Other electrode clusters revealing *Affective Category x Stimulation* interaction effects in the 200-600 ms time window during encoding (Study 1).**

For Cluster 2, a main effect of *Affective Category* was observed, F(1,25) = 6.39, p = .02, *η*_p_^2^ = 0.20, but no main effect of *Stimulation* was found, F(1,25) = 1.00, p = .32, *η*_p_^2^ = 0.04. The *Affective Category x Stimulation* interaction reached significance, F(1,25) = 13.22, p = .001, *η*_p_^2^ = 0.35 (BF_10_= 1.09). Subsequent analysis revealed that no differences between unpleasant and neutral images under sham emerged, t(26) = -1.01, p = .32, but neutral images under taVNS produced larger amplitudes than unpleasant ones, t(26) = 3.36, p = .003. Furthermore, in the absence of differences between groups for neutral images, t(26) = -0.39, p = .69, unpleasant images under sham produced larger amplitudes than under taVNS, t(26) = 2.50, p = .02.

For Cluster 3, no main effect of *Affective Category* was observed, F(1,25) = 0.97, p = .76, *η*_p_^2^ = 0.0, but the main effect of *Stimulation*, F(1,25) = 8.15, p = .32, *η*_p_^2^ = 0.04, and the *Affective Category x Stimulation* interaction reached significance, F(1,25) = 10.97, p = .003, *η*_p_^2^ = 0.31 (BF_10_= 0.45). Follow-up analysis revealed no differences between unpleasant and neutral images under sham, t(26) = -1.87, p = .07, but neutral images under taVNS produced larger amplitudes than unpleasant ones, t(26) = 2.43, p = .023. Furthermore, larger amplitudes for neutral images encoded under taVNS compared to sham stimulation were observed, t(26) = -3.94, p < .001, but no differences were found between stimulation conditions for unpleasant images, t(26) = -1.50, p = .14.

For Cluster 4, no main effect of *Affective Category*, F(1,25) = 1.29, p = .27, *η*_p_^2^ = 0.05, or *Stimulation* was observed, F(1,25) = 0.01, p = .95, *η*_p_^2^ = 0.0, but the interaction between *Affective Category* and *Stimulation* reached significance, F(1,25) = 11.73, p = .002, *η*_p_^2^ = 0.32 (BF_10_= 0.91). Follow-up analysis revealed no differences between unpleasant and neutral images under sham, t(26) = -1.68, p = .11, but neutral images under taVNS produced larger amplitudes than unpleasant ones, t(26) = 2.80, p = .001. Nevertheless, no differences between conditions emerged for neutral, t(26) = -1.46, p = .16, or unpleasant images, t(26) = 1.69, p = .10.

**S4. Cluster-based permutation tests revealing *Affective Category* effects during encoding (Study 2).**

In the early time window (200 - 600 ms), two clusters over posterior regions surpassed the cluster mass of 1043.5 (Cluster 1: mass = 5805.8, time window = 436 - 600 ms, sensors 37, 41, 42, 46, 47, 51, 52, 53, 54, 55, 59, 60, 61, 62, 65, 66, 67, 70, 71, 72, 75, 76, 77, 78, 79, 83, 84, 85, 86, 87, 90, 91, 91, 93, 96, 97, 98, 101, 102, and 103); Cluster 2: mass = 4155.6 , time window = 384 - 600 ms, sensors =49, 56, 57, 63, 64, 69, 82, 83, 88, 89, 90, 94, 95, 96, 99, 100, 101, 107, and 113). Figure S2 shows the mean amplitudes for each condition for the significant clusters and time windows.

Analysis on Cluster 1 revealed a main effect of *Affective Category,* F(1,59) = 4.14, p < .046, *η*_p_^2^ = 0.06, but no effects of *Stimulation,* F(1,59) = 0.006, p = .94, *η*_p_^2^ = 0.00, or *Affective Category x Stimulation* interaction*,* F(1,59) = 2.78, p = .10, *η*_p_^2^ = 0.04. Although there was no significant interaction, we conducted t-tests to explore the differences unpleasant and neutral images for the taVNS and sham group, separately. Subsequent t-tests revealed significant differences between unpleasant and neutral scenes in the taVNS group, t(30) = -2.46, p =.02, but not in the sham group, t(29) = -0.28, p = .78.

For Cluster 2, a main effect of *Affective Category* was observed, F(1,59) =14.26, p <.001, *η*_p_^2^ = 0.19. No effect of *Stimulation*, F(1,59) = 0.41, p = .52, *η*_p_^2^ = 0.07, or *Affective Category x Stimulation* interaction emerged, F(1,59) = .304, p = 0.58, *η*_p_^2^ = 0.005. Despite the lack of a significant interaction, we conducted t-tests to explore the differences unpleasant and neutral images for the taVNS and sham group, separately. Subsequent t-tests revealed significant differences between unpleasant and neutral scenes under taVNS, t(30) = 2.42, p =.021, but not under sham stimulation, t(29) = 2.89, p = .007.

In the late time window (600 - 1200 ms) three posterior electrode clusters surpassed the cluster mass of 836.5 (Cluster 1: mass= 61211.9, time window = 600-1192 ms, sensors = 37, 41, 42, 46, 47, 51, 52, 53, 54, 55, 59, 60, 61, 62, 65, 66, 67, 70, 71, 72, 75, 76, 77, 78, 79, 83, 84, 85, 86, 87, 90, 91, 92, 93, 96, 97, 98, 101, 102, and 103; Cluster 2: mass = 1937.2 , time window = 600 - 1004 ms, sensors = 88, 89, 94, 95, 99, 100, 107, 108, and 113; Cluster 3: mass = 2577.6, time window = 600 - 992, sensors= 49, 56, 57, 63, and 64)

For Cluster 1, results revealed a main effect of *Affective Category,* F(1,25) =35.24, p < .001, *η*_p_^2^ = 0.37, but no main effect of *Stimulation,* F(1,59) = 0.01, p = .91, *η*_p_^2^ = 0.00, or interaction effect, *Affective Category x Stimulation:* F(1,59) =0.715, p = .40, *η*_p_^2^ = 0.01. Although no significant interaction effect were observed, we conducted t-tests to explore the differences between unpleasant and neutral images for the taVNS and sham condition, separately. Subsequent t-tests revealed significant differences between unpleasant and neutral scenes in both the taVNS, t(30) = -4.80, p <.001, and sham group, t(29) = -3.96, p < .001.For Cluster 2, a main effect of *Affective Category* was found*,* F(1,59) = 37.77, p < .001, *η*_p_^2^ = 0.39, but no *Stimulation,* F(1,59) = 0.006, p = .93, *η*_p_^2^ = 0.0, or *Affective Category x Stimulation* interaction effect*,* F(1,25) =0.24, p = .62, *η*_p_^2^ = 0.004. Subsequent t-tests revealed significant differences between unpleasant and neutral images in both the taVNS t(30) = -5.19, p < .001, and sham group, t(29) = -3.92, p <.001. For Cluster 3, a main effect of *Affective Category* was found*,* F(1,59) = 39.09, p < .001, *η*_p_^2^ = 0.40, but no main effect of *Stimulation,* F(1,59) = 0.005, p = .94, *η*_p_^2^ = 0.0, or *Affective Category x Stimulation* interaction effect*,* F(1,25) =0.19, p = .67, *η*_p_^2^ = 0.003. Subsequent t-tests revealed significant differences between unpleasant and neutral images in both the taVNS t(30) = -5.34, p < .001, and sham group, t(29) = -3.95, p <.001.

Altogether, the expected larger LPP amplitudes for emotional compared to neutral scenes were observed in an earlier (Cluster 1, 436-600 ms) and later time window (Cluster 1, 600 - 1192 ms and Cluster 2: 600 - 1004). However, for the early time window, the effects were mostly driven by the taVNS group (see section S8 for follow-up analysis on the sham group).

**
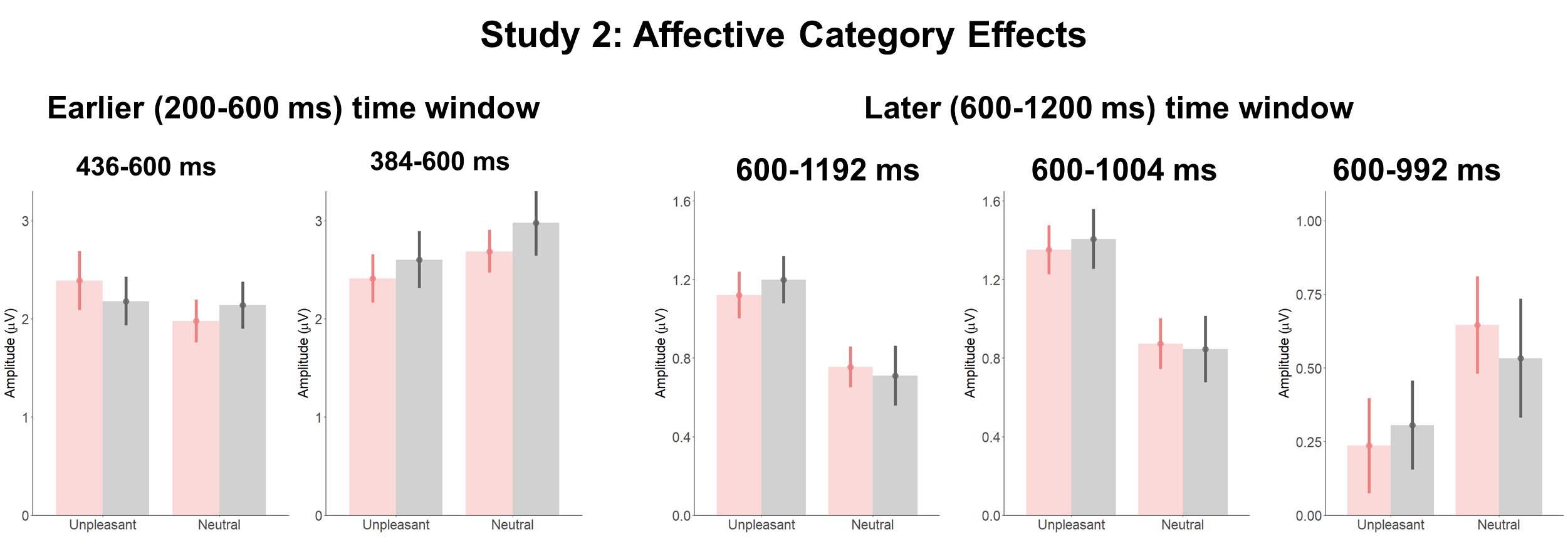
**

Figure S2. Mean averaged ERPs during the significant time windows and sensor clusters, for the earlier (left) and later (right) time windows of the *Affective Category* effects.

**S5. Cluster-based permutation tests revealing *Stimulation* effects during encoding (Study 2).**

Neither in the early (200 - 600 ms) nor in the later (600 -1200 ms) time window, a cluster surpassed the critical cluster masses of 909 and 803.5, respectively.

**S6. Cluster-based permutation test revealing *Memory* effects during retrieval (Study 2).**

Due to the considerable *Memory* effects we used a more conservative sensor and cluster threshold (p <.01). Under these conditions, three clusters surpassed the critical cluster mass of 842 (Cluster 1: mass = 41174.1, time window = 428 - 868 ms, sensors: 5, 6, 7, 11, 12, 13, 19, 20, 23, 24, 27, 28, 29, 30, 31, 32, 36, 37, 38, 42, 43, 44, 45, 48, 49, 50, 51 ,52, 53, 54, 55, 56, 57, 58, 59, 60, 61, 62, 63, 64, 65, 67, 68, 69, 70, 72, 73, 74, 77, 78, 79, 80, 81, 82, 85, 86, 87, 92, 93, 104, 105, 106, 110, 111, 112, 128, and 129; Cluster 2: mass = 5172.3, time window = 472 - 688 ms, sensors = 1, 107, 113, 114, 115, 119, 120, 121, 122, 125, and 126; Cluster 3; mass = 1002.3, time window: 916-980, sensors: 5, 6, 7, 11, 12, 13, 18, 19, and 23).

Analysis on Cluster 1 revealed a main effect of *Affective Category,* F(1,50) = 27.78, p < .001 *η*_p_^2^ = 0.36, but no main effect of *Stimulation,* F(1,50) = 0.21, p = 0.65, *η*_p_^2^ = 0.004. The main effect of *Memory* reached significance, F(1,50) = 16.89, p < 0.001, *η*_p_^2^ = 0.25. Neither, the *Stimulation x Affective Category* interaction, F(1,50) = 0.25, p = 0.69, *η*_p_^2^ = 0.005, the *Affective Category x Memory* interaction, F(1,50) = 3.52, p = 0.06, *η*_p_^2^ = 0.06, nor the *Affective Category x Memory x Stimulation* interaction, F(1,50) = 2.04, p = 0.15, *η*_p_^2^ = 0.04 reached significance. However, a *Group x Memory* interaction emerged, F(1,50) = 4.32, p = 0.043, *η*_p_^2^ = 0.08 (see Figure S3). Follow-up t-test revealed significant Old/New differences in the taVNS group for unpleasant images, t(23)= 4.92, p < .001, but not for neutral ones, or for the sham group (*p*s>0.12).

Analysis on Cluster 2 revealed a main effect of *Affective Category,* F(1,50) = 45.62, p < .001 *η*_p_^2^ = 0.47, but no main effect of *Stimulation,* F(1,50) = 1.44, p = 0.23, *η*_p_^2^ = 0.028. The main effect of *Memory* reached significance, F(1,50) = 21.91, p < 0.001, *η*_p_^2^ = 0.30. Neither, the *Stimulation x Affective Category* interaction, F(1,50) = 2.85, p = 0.09, *η*_p_^2^ = 0.05, the *Group x Memory* interaction, F(1,50) = 1.37, p = 0.24, *η*_p_^2^ = 0.02, nor the *Affective Category x Memory x Stimulation* interaction, F(1,50) = 0.18, p= 0.67, *η*_p_^2^ = 0.004 reached significance. However, an *Affective Category x Memory* interaction emerged, F(1,50) =4.60, p = 0.037, *η*_p_^2^ = 0.08 (see Figure S3). Follow-up t-test showed that old/new differences only emerged for unpleasant images in both the taVNS, t(23)=-3.53, p = .001, and sham group, t(27) = 4.22, p < .001, but not for neutral ones, (*p*s>0.08).

Analysis on Cluster 3 revealed a main effect of *Memory,* F(1,50) =12.03, p < .001 *η*_p_^2^ = 0.19, but no other effects reached significance (Fs < 3.64, ps < 3.64; see Figure S3).


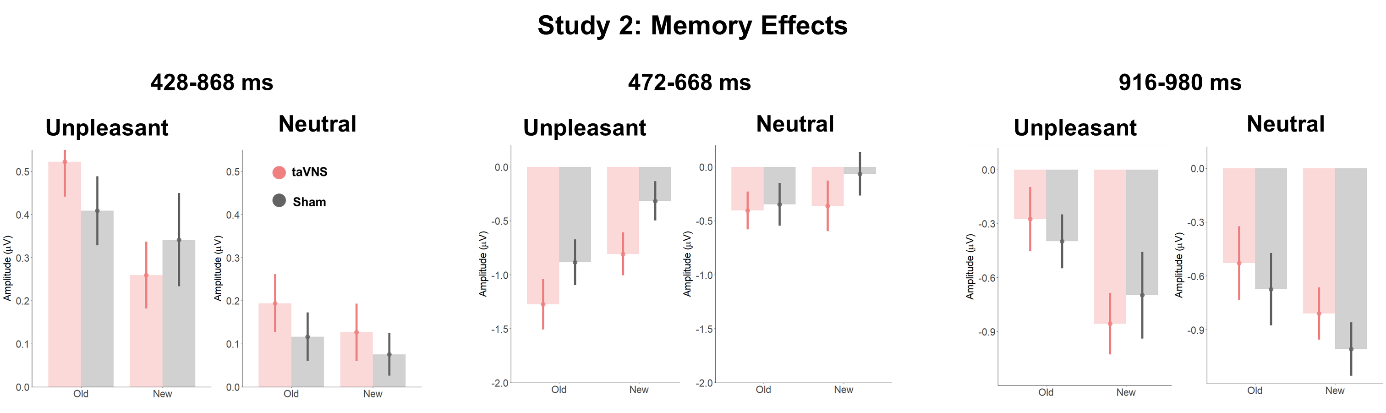
Altogether, we replicated the late ERP Old/New effect as indicated by larger amplitudes for correctly identified *old* compared to *new* scenes (Cluster 1, 428-868 ms, Cluster 3, 916-980 ms).

Figure S3. Mean averaged ERPs during the significant time windows and sensor clusters, for the earlier (left) and later (right) time windows of the *Memory* effects.

**S7. Cluster-based permutation tests revealing *Memory x Affective Category* effects during retrieval (Study 2).**

For the *Memory x Affective Category* interaction effects, two central and frontal clusters surpassed the critical cluster mass of 1386 (Cluster 1: mass = 6389.8, time window = 492 - 680 ms, sensors: 5, 66, 7, 13, 29, 30, 31, 36, 37, 42, 52, 53, 54, 55, 60, 61, 62, 67, 71, 72, 76, 77, 78, 79, 80, 84, 85, 86, 87, 92, 93, 104, 105, 106, 112, and 129; Cluster 2: mass = 3334.7 , time window = 456 - 820 ms, sensors = 32, 33, 34, 38, 39, 40, 43, 44, 48, 49, 127, and 128).

Analysis on Cluster 1 revealed a main effect of *Affective Category,* F(1,50) = 72.32, p < .001 *η*_p_^2^ = 0.61, but no main effect of *Stimulation,* F(1,50) = 0.15, p = 0.69, *η*_p_^2^ = 0.003. The main effect of *Memory* reached significance, F(1,50) = 41.41, p < 0.001, *η*_p_^2^ = 0.45. Neither, the *Stimulation x Affective Category* interaction, F(1,50) = 0.20, p = 0..88, *η*_p_^2^ = 0.00, the *Group x Memory* interaction, F(1,50) = 1.81, p = 0.18, *η*_p_^2^ = 0.03, nor the *Affective Category x Memory x Stimulation* interaction, F(1,50) = 0.28, p= 0.59, *η*_p_^2^ = 0.006 reached significance. However, an *Affective Category x Memory* interaction emerged, F(1,50) =12.0, p = .001, *η*_p_^2^ = 0.19 (see Figure S4). Follow-up t-test revealed Old/New differences for unpleasant images in both the taVNS, t(23)= 6.02, p = .001, and sham group, t(27) = 3.79, p < .001, but not for neutral ones, (*p*s>0.23).

Analysis on Cluster 2 revealed a main effect of *Affective Category,* F(1,50) = 53.24, p < .001 *η*_p_^2^ = 0.51, but no main effect of *Stimulation,* F(1,50) = 0.04, p = 0.83, *η*_p_^2^ = 0.00. The main effect of *Memory* reached significance, F(1,50) = 13.51, p < 0.001, *η*_p_^2^ = 0.21. Neither, the *Stimulation x Affective Category* interaction, F(1,50) = 3.93, p = 0.053, *η*_p_^2^ = 0.07, the *Group x Memory* interaction, F(1,50) = 0.70, p = 0.41, *η*_p_^2^ = 0.01, nor the *Affective Category x Memory x Stimulation* interaction, F(1,50) = 0.15, p= 0.69, *η*_p_^2^ = 0.003 reached significance. However, an *Affective Category x Memory* interaction emerged, F(1,50) =7.12, p = .012, *η*_p_^2^ = 0.13 (see Figure S4). Follow-up analysis revealed less negative amplitudes for new compared to old unpleasant images in both the taVNS, t(23)= -2.67, p = .01, and sham group, t(27) = -3.92, p < .001, but not for neutral ones, (*p*s>0.69).

Altogether, we found an enhanced ERP Old/New effect for unpleasant compared to
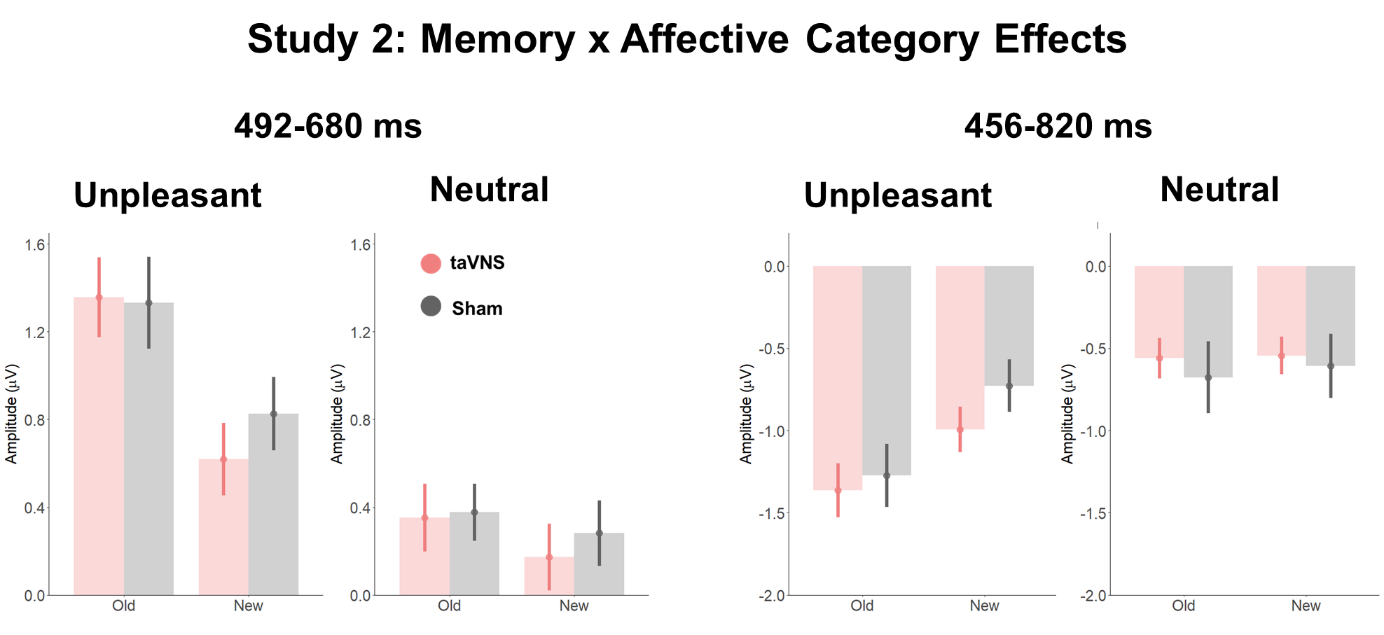
neutral scenes (Cluster 1, 492 - 680 ms), which is in line with prior findings.

Figure S3. Mean averaged ERPs during the significant time windows and sensor clusters, for the earlier (left) and later (right) time windows of the *Memory x Affective Category* effects.

**S8. Cluster-based permutation tests revealing *Affective Category* effects during encoding for the sham condition in the early time window of the LPP (Study 1 and Study 2).**

The main effect of *Affective Category* (see sections S1 and S4) observed in the earlier time window of the LPP in Study 1 and Study 2 seem to be mostly driven by larger differences between unpleasant and neutral images in the taVNS condition. Although the *Affective Category* x *Stimulation* interaction was not significant, only at trend level, this is supported by post-hoc testing (Clusters 1 and 2 in Study 1; Cluster 1 in Study 2). To directly test whether the expected larger LPP amplitudes for unpleasant compared to neutral images are also observed in the sham condition, we analyzed the main effects of *Affective Category* for the sham condition alone in both Study 1 and Study 2.

For Study 1, results revealed one cluster with a main effect of category (time window: 384-600ms, sensors: 17, 21, 25, 26, 32, 33, 35, 38, 39, 40, 41, 44, 45, 46, 50, 57, and 128). Contrary to what it was expected, the significant cluster depicted larger positivity for neutral compared to unpleasant images, t(26) = 3.08, p = .005.

For Study 2, results showed main effects of affective category in 3 different clusters (Cluster 1: time window: 428-600, sensors: 5, 6, 7, 12, 13, 20, 28, 29, 30, 31, 35, 36, 37, 41, 42, 47, 53, 54, 55, 79, 80, 87, 104, 105, 106, 111, 112, 118, and 129; Cluster 2: time window: 384-596 ms, sensors: 69, 70, 73, 74, 75, 81, 82, 83, 88, 89, 90, 94, 95, 96, 99, 100, 101, and 108; Cluster 3: time window: 200-368, sensors: 1, 2, 3, 4, 8, 9, 10, 11, 14, 15, 16, 17, 113, 114, 115, 117, 118, 119, 120, 121, 122, 123, 124, 125, and 126). Cluster 1 and Cluster 3, revealed larger amplitudes for unpleasant compared to neutral images (Cluster 1: t(29) = -3.21, p = .003; Cluster 3: t(29) = -2.97, p = .006). Whereas Cluster 2 showed the opposite pattern, t(29) = 3.41, p = .001.

These findings indicate that the Affective Category effects (larger amplitudes for emotional compared to neutral pictures) were less prominent in the early time window of the LPP in the sham condition. However, the fact that taVNS particularly facilitated affective discrimination in the early LPP time window may suggest that vagal stimulation may lead to an earlier enhanced LC-mediated neural gain (e.g., Aston-Jones and Cohen, 2005; Nieuwenhuis et al., 2005) that may promote enhanced attentional processing of task (Ventura-Bort et al., 2018) or motivationally relevant events (Maraver et al., 2020; Ventura-Bort et al., 2021) at the cost of less relevant information (in line with GANE model by Mather et al., 2016).

**
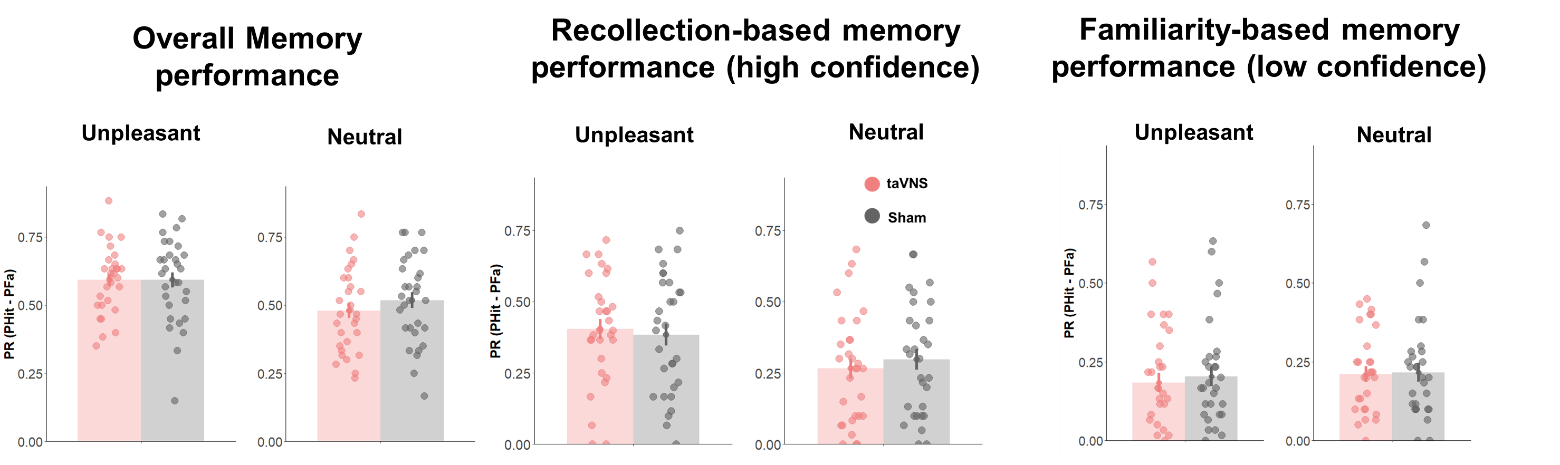
S9. Visual depiction of the memory performance, overall and split by familiarity and recollection.**

Figure S4. Overall (left) memory performance, for recollection-related (middle) and familiarity-related (right) judgments. Depiction of behavioral performance for unpleasant and neutral images of Study 1. Error bars represent standard error. Bar plots represent mean values. Dots indicate individual scores.


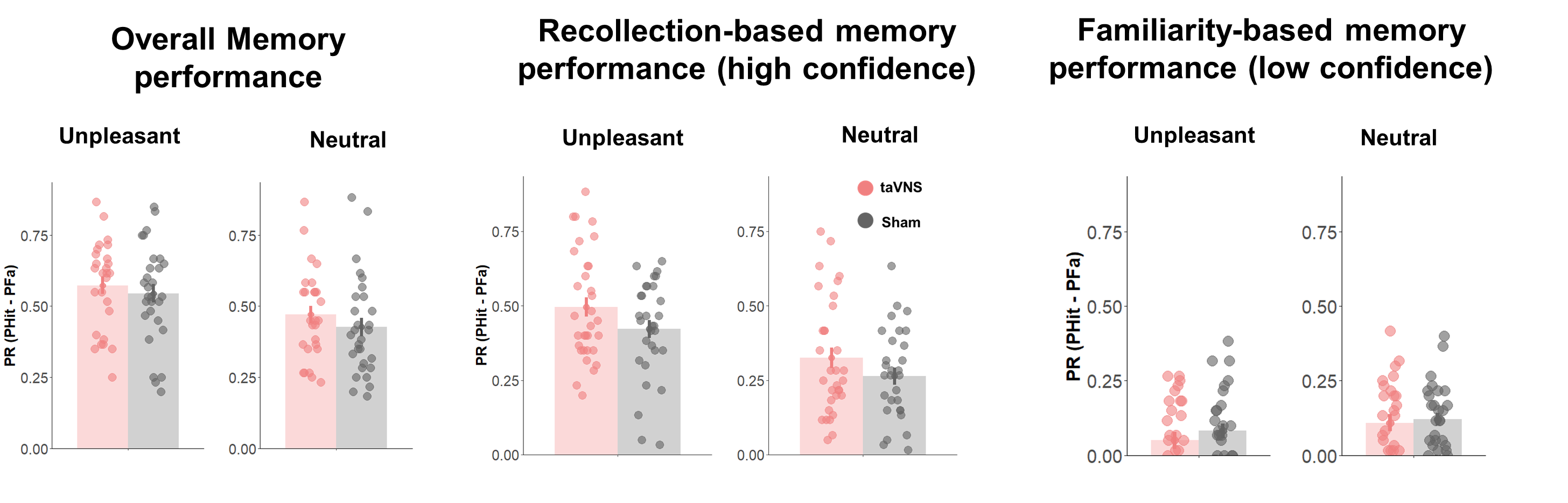


Figure S5. Overall (left) memory performance, for recollection-related (middle) and familiarity-related (right) judgments. Depiction of behavioral performance for unpleasant and neutral images of Study 2. Error bars represent standard error. Bar plots represent mean values. Dots indicate individual scores.
